# The intrinsic expression of NLRP3 in Th17 cells promotes their protumor activity and conversion into Tregs

**DOI:** 10.1038/s41423-025-01281-y

**Published:** 2025-04-07

**Authors:** Théo Accogli, Christophe Hibos, Lylou Milian, Mannon Geindreau, Corentin Richard, Etienne Humblin, Romain Mary, Sandy Chevrier, Elise Jacquin, Antoine Bernard, Fanny Chalmin, Catherine Paul, Berhard Ryffel, Lionel Apetoh, Romain Boidot, Mélanie Bruchard, François Ghiringhelli, Frédérique Vegran

**Affiliations:** 1 INSERM, Dijon, France; 2 University of Burgundy, Dijon, France; 3 Unité de Biologie Moléculaire—Department of Biology and Pathology of Tumors, Georges-Francois Leclerc Cancer Center-UNICANCER, Dijon, France; 4 Cancer Biology Transfer Platform, Georges-Francois Leclerc Cancer Center-UNICANCER, Dijon, France; 5 LIIC, EA7269, Université de Bourgogne Franche Comté, Dijon, France; 6 Immunology and Immunotherapy of Cancer Laboratory, EPHE, PSL Research University, Paris, France; 7 Laboratory of Experimental and Molecular Immunology and Neurogenetics (INEM), UMR 7355 CNRS-University of Orleans, Orléans, France; 8 Brown Center for Immunotherapy, Indiana University Melvin and Bren Simon Comprehensive Cancer Center, Indiana University School of Medicine, Indianapolis, IN USA; 9 Genetic and Immunology Medical Institute, Dijon, France; 10 Department of Medical Oncology, Centre Georges-François Leclerc, Dijon, France

**Keywords:** Th17 cells, Tregs, NLRP3, Tumor microenvironment, Cancer Immunology, Tumour immunology, Adaptive immunity

## Abstract

Th17 cells can perform either regulatory or inflammatory functions depending on the cytokine microenvironment. These plastic cells can transdifferentiate into Tregs during inflammation resolution, in allogenic heart transplantation models, or in cancer through mechanisms that remain poorly understood. Here, we demonstrated that NLRP3 expression in Th17 cells is essential for maintaining their immunosuppressive functions through an inflammasome-independent mechanism. In the absence of NLRP3, Th17 cells produce more inflammatory cytokines (IFNγ, Granzyme B, TNFα) and exhibit reduced immunosuppressive activity toward CD8+ cells. Moreover, the capacity of NLRP3-deficient Th17 cells to transdifferentiate into Treg-like cells is lost. Mechanistically, NLRP3 in Th17 cells interacts with the TGF-β receptor, enabling SMAD3 phosphorylation and thereby facilitating the acquisition of immunosuppressive functions. Consequently, the absence of NLRP3 expression in Th17 cells from tumor-bearing mice enhances CD8 + T-cell effectiveness, ultimately inhibiting tumor growth.

## Introduction

Th17 cells are characterized by their capacity to secrete IL-17A, IL-17F, IL-21, IL-22, and CCL20. Th17 cells develop from naïve CD4+ T cells in the presence of TGF-β and IL-6. Their initiation is driven by TCR activation, which induces the expression of the transcription factors IRF4 and BATF, along with IL-6-dependent STAT3 activation and TGF-β. These molecular pathways collectively drive the expression of the master transcription factor Retinoic acid-related Orphan Receptor-*γt* (*RORγt)* [1] to establish the Th17 cell program. While Th17 cells are rare in healthy mice, they expand in the blood, bone marrow, and spleen of tumor-bearing mice and in human cancers, such as melanoma, prostate cancer, fibrosarcoma, and advanced head and neck cancer [2–4]. Despite their prevalence in these contexts, the role of Th17 cells in cancer immunity remains controversial. Although the molecular basis for these discrepancies is elusive, evidence suggests that Th17 cells may adopt regulatory or inflammatory (also called pathogenic) functions depending on cytokines microenvironment [5].

Th17 cells appear to be plastic cells and can transdifferentiate into Th1 or Treg cells. The conversion of Th17 cells into Tregs has been studied in various contexts. Th17 cells can transdifferentiate into IL-10-producing Tregs, termed Tr1 cells, during inflammation resolution [6]. Moreover, Th17 cells could be a source of Tregs in allogenic heart transplantation models [7]. In human cancers, Foxp3+ RORγt + IL17+ cells can be found at tumor sites. Th17-to-Treg transdifferentiation in tumors has also been documented in mice [8], but the mechanisms involved are still poorly understood.

The NLRP3 protein, a member of the Nod-Like Receptor (NLR) family, has been extensively studied in myeloid cells. In response to danger signals, the cytoplasmic NLRP3 protein interacts with the adapters Apoptosis-associated Speck-like protein (ASC) and Caspase-1 to form a macromolecular complex called the NLRP3 inflammasome. This complex leads to the cleavage and activation of the Caspase-1 precursor and induces the cleavage and maturation of IL-1β and IL-18, which are involved in the induction of inflammation [9]. Recently, inflammasome-independent roles for NLRP3 have been described in lymphoid cells. Its expression has been detected in human and murine CD4+ T cells [10]. Previously, we reported that NLRP3 expression is induced by STAT5 in all CD4+ T cells under the autocrine effect of IL-2 produced after TCR activation. In Th2 lymphocytes, we discovered that NLRP3 acts as a transcription factor via interactions with IRF4 [11].

In this study, we investigated the role of NLRP3 in Th17 cells in the context of cancer. We found that NLRP3 expression in Th17 cells is essential for maintaining their immunosuppressive functions. Loss of NLRP3 led to increased production of inflammatory cytokines (IFNγ, Granzyme B, TNFα) and impaired immunosuppression of CD8+ cells. Additionally, NLRP3-deficient Th17 cells fail to transdifferentiate into Treg-like cells. Mechanistically, NLRP3 stabilizes SMAD3 phosphorylation via its interaction with the TGF-β receptor, enabling the acquisition of immunosuppressive functions. Therefore, in tumor-bearing mice, the absence of NLRP3 in Th17 cells enhances CD8+ cell activity and inhibits tumor growth.

## Results

### NLRP3 plays a role in Th17 cell biology

In a previous study, we demonstrated the involvement of NLRP3 in Th2 differentiation [11]. To investigate the role of NLRP3 in CD4+ T cells, we generated *Nlrp3* flox mice via the Cre–loxP system to target exon 4 of the *Nlrp3* gene in the mouse genome. Crossing these mice with CD4cre mice led to Cre recombinase-mediated removal of the 4^th^ exon of *Nlpr3* in CD4+ T cells, resulting in a premature stop codon and loss of NLRP3 in these cells (Supplementary Fig. 1a). We confirmed NLRP3 deficiency through immunofluorescence staining and Western Blot analysis of CD4+ T cells isolated from *Nlrp3*^flox/flox^CD4^Cre^ mice (henceforth referred to as CD4^*Nlrp3-/-*^) by differentiating them into Th2 cells and comparing them with Th2 cells differentiated from WT mice and completely deficient in *Nlrp3* mice (Supplementary Fig. 1b, c). The absence of NLRP3 expression was accompanied by reduced levels of *Gata-3*, a Th2-specific transcription factor, and *Il4*, the main cytokine produced by these cells (Supplementary Fig. 1d). To characterize these mice further, we assessed the frequency of double-positive (CD4+CD8+), CD4+, and CD8+ cells in the thymus and spleen and found no significant differences. Similarly, the proportions of naïve, central memory, and effector memory CD8+ T cells were comparable between the CD4^*Nlrp3-/-*^ mice and their controls. Finally, cytokine production by splenic CD8+ cells under steady-state conditions was also unaffected (Supplementary Fig. 1e–h).

**Fig. 1:**
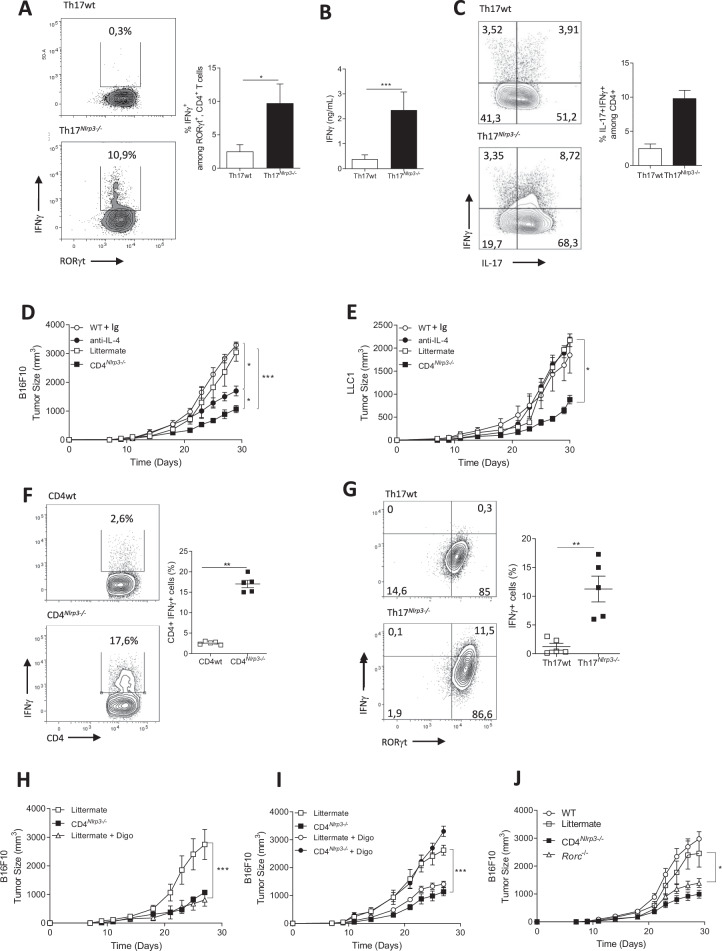
NLRP3 is involved in Th17 differentiation. **A** IFNγ production detected by cytometry in Th17 cells differentiated in vitro with TGF-β and IL-6 for 72 h from naïve CD4 T cells isolated from CD4^*Nlrp3-/-*^ (Th17^*Nlrp3-/-*^) mice and their littermate controls (Th17wt). **B** Quantification of IFNγ detected by ELISA in the same samples as in (**A**). **C** IFNγ and IL-17 production was detected by flow cytometry under the same conditions as in (**A**). Tumor growth (*n* = 5) of B16F10 (**D**) and LLC1 (**E**) cells in C57BL/6 WT mice treated with an IL-4 blocking antibody (anti-IL-4) or a control Ig and in CD4^*Nlrp3-/-*^ mice and their littermate controls. IFNγ production detected by cytometry in CD4+ T cells (**F**) and Th17 cells (CCR6 + RORγt+Foxp3-) (**G**) from B16F10 tumors. B16F10 tumor growth (n = 5) in CD4^*Nlrp3-/-*^ mice and controls treated with or without digoxin (Digo) (**H**, **I**) and in C57Bl6 WT, *Rorc-/-*, and CD4^*Nlrp3-/-*^ mice and their respective controls (**J**). The data shown are representative of 3 independent in vivo experiments or 5 independent in vitro experiments. Statistical significance was determined by 2-way ANOVA and Tukey’s multiple comparison tests (**D**, **E**, **H**–**J**) or the Mann‒Whitney test (**A**‒**C**, **F**, **G**). * <0.05, **<0.01, ***<0.005

To examine the impact of NLRP3 on CD4+ T-cell behavior, we isolated naïve CD4+ T cells from CD4^*Nlrp3-/-*^ mice and littermate controls (corresponding to *Nlrp3*^flox/flox^CD4^WT^ mice.) and differentiated them in vitro into Th1, Th2, Th17 (in the presence of TGF-β and IL-6), and Tregs. Master transcription factor and cytokine expression were analyzed via RT‒qPCR. While Th2 differentiation was impaired, as evidenced by the reduced expression of *Gata3* and *Il4* (Supplementary Fig. 2), we also observed increases in both the mRNA and protein levels of IFNγ during Th17 differentiation in the absence of NLRP3 (Supplementary Fig. 2 and Fig. 1A–C). Importantly, *Nlrp3* deficiency did not affect Th17 cell proliferation (Supplementary Fig. 3a).

We studied the growth of subcutaneous tumors (B16F10 melanoma and LLC1 lung cancer) in CD4^*Nlrp3-/-*^ and control mice. Tumor growth was significantly reduced in the CD4^*Nlrp3-/-*^ mice in both models (Fig. 1D, E). Notably, the growth delay was similar to that observed in mice completely deficient in *Nlrp3* (Supplementary Fig. 3b, c). Administration of an IL-4 blocking antibody did not significantly affect LLC1 tumor growth and had a minor effect on B16F10 growth (Fig. 1D, E), suggesting that the delay in tumor growth in CD4^*Nlrp3-/-*^ mice is not solely due to impaired Th2 differentiation and IL-4 production. *Nlrp3* was rapidly induced during Th17 cell differentiation (Supplementary Fig. 3d). At the protein level, nearly 80% of Th17 cells expressed NLRP3 after 24 h of differentiation (Supplementary Fig. 3e, f).

The canonical role of NLRP3 involves its participation in the inflammasome and IL-1β/IL-18 secretion [9]. Compared with that in control mice, IL-1β production was equivalent in tumors from CD4^*Nlrp3-/-*^ mice, whereas IL-1β production was completely abolished in tumors from *Nlrp3* total knockout mice (Supplementary Fig. 3g). Unlike macrophages, CD4+ T cells from littermate or CD4^*Nlrp3-/-*^ mice do not produce IL-1β or IL-18 upon ATP stimulation, as reported in the literature [12] (Supplementary Fig. 3h). Using data from RNA-Seq experiments conducted a few years ago [13], we analyzed the expression of genes encoding caspases, TLR family members, and inflammasome-produced cytokines. We did not detect any expression of *Caspase-1* or *Tlr4* in CD4+ T cells across all subtypes after 24 h of differentiation (Supplementary Fig. 3i, j). A recent study demonstrated that during TCR activation, CD4+ T cells produce IL-1α through an inflammasome mechanism that induces Caspase-8 activation [14]. Our RNA-Seq data confirmed this gene induction in our experiments (Supplementary Fig. 3i, k). However, blocking inflammasome-related pathways via either Caspase-1 (ZVAD) or Caspase-8 (ZIETD) inhibitors, MCC950 (which inhibits inflammasome assembly) or Anakinra (which targets IL-1RA) during in vitro Th17 differentiation (in the presence of TGF-β and IL-6), did not replicate the *Ifng* upregulation observed in *Nlrp3-*deficient Th17 cells (Supplementary Fig. 3l). Similarly, CD4+ T cells isolated from *Asc-*, *Ice-*, *Il1r1-* and *Il18r*-deficient mice did not exhibit increased *Ifng* expression when differentiated into Th17 cells (Supplementary Fig. 3m). Furthermore, treating mice with MCC950 had no effect on tumor growth in either the B16F10 or LLC1 model (Supplementary Fig. 3n, o), indicating that the inflammasome has no discernible effect on the growth of these tumors.

Flow cytometry analysis of CD4+ Tumor-Infiltrating Lymphocytes (TILs) and draining lymph nodes from B16F10 tumor-bearing mice revealed increased IFNγ production in CD4^*Nlrp3*-/-^ mice compared with control mice at both the mRNA (Supplementary Fig. 4a) and protein levels (Supplementary Fig. 4b and Fig. 1F). Specifically, Th17 cells (characterized as CCR6^+^RORγt^+^FOXP3^-^) infiltrating B16F10 tumors produced more IFNγ at both the mRNA (Supplementary Fig. 4c) and protein levels (Fig. 1G) in CD4^*Nlrp3*-/-^ mice than in control mice. Treatment with digoxin, a RORγt inhibitor that depletes Th17 cells [15], reduced B16F10 (Fig. 1H and Supplementary Fig. 4d) and LLC1 (Supplementary Fig. 4e) tumor growth in control mice to levels similar to those observed in CD4^*Nlrp3-/-*^ mice. In contrast, the antitumor effect of CD4^*Nlrp3-/-*^ was abolished by Digoxin, suggesting that the effect is entirely dependent on Th17 cells (Fig. 1i). These data were corroborated in RORγt-deficient mice (Fig. 1j and Supplementary Fig. 4f).

Collectively, these results suggest that Nlrp3 influences Th17 cell function in cancer and reduces tumor growth in an inflammasome-independent manner.

### NLRP3 deficiency favors a Th17 inflammatory profile

**Fig. 2:**
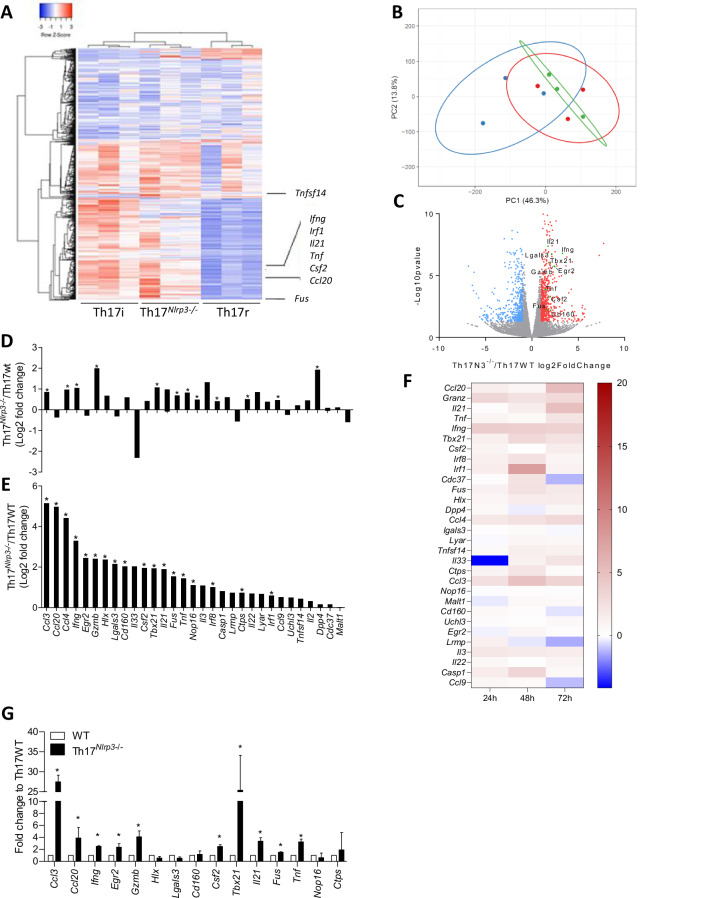
NLRP3 deficiency favors a Th17 inflammatory profile. WT inflammatory Th17 cells (Th17i), WT regulatory Th17 cells (Th17r), and *Nlrp3*-deficient regulatory Th17 cells (Th17^*Nlrp3-/-*^) were differentiated in vitro for 72 h (**A–C**, **E**) or 24 h (**D**). **A** Heatmap representing hierarchical clustering of RNA-seq data. **B** Principal component analysis (PCA) of the same data as in (**A**). Blue: triplicates of regulatory WT Th17 cells; red: triplicates of inflammatory WT Th17 cells; green: triplicates of regulatory Th17^*Nlrp3-/-*^ cells. **C** Volcano plot showing the distribution of differentially expressed genes from the same data as in (**A**), with upregulated genes in red, downregulated genes in blue, and the inflammatory gene signature in green. Differential expression of genes associated with the inflammatory profile of Th17 cells according to the RNA-seq data after 24 h (**D**) and 72 h (**E**) of in vitro differentiation. **F** Heatmap representing the differential expression of the Th17 inflammatory profile evaluated by RT‒qPCR over a time course of 24 h, 72 h and 7 days of in vitro differentiation. **G** Differential expression of the inflammatory profile of Th17 cells isolated from B16F10 tumors from CD4^*Nlrp3-/-*^ mice and their controls was evaluated via RT‒qPCR. Th17 cells were defined as CD4 + CCR6+Foxp3- cells. The data presented are pooled from 3 independent experiments. **A** Unsupervised hierarchical clustering was performed via Gene Cluster 3.0 software, and the results were visualized with Treeview viewer. Gene expression was normalized and mean-centered. Hierarchical clustering was conducted via the correlation measure and complete linkage analysis. **D**, **E** Cuffdiff analysis. **G** Statistical significance was determined via 2-way ANOVA. Asterisks indicate significant differences

Our results demonstrate that Th17 cells lacking *Nlrp3* exhibit increased IFNγ production. To explore the molecular state of Th17 cells in the context of *Nlrp3* deficiency, we performed mRNA profiling of differentiated Th17 cells in vitro via RNA-seq (Th17 cells differentiated from *Nlrp3*-deficient naïve T cells are referred to as *Nlrp3*-deficient Th17 cells hereafter). We compared the gene expression profiles of WT or *Nlrp3*-deficient Th17 cells under 2 different conditions: with TGF-β1 and IL-6 to generate Th17 cells with a regulatory profile and with IL-1β, IL-6, and IL-23 (without TGF-β) to induce inflammatory Th17 cells. Our data revealed that *Nlrp3-*deficient Th17 cells differentiated with TGF-β1 and IL-6 presented a significantly altered transcriptional profile compared with WT Th17 cells, with 759 differentially expressed genes (FDRs ≤ 0.05). Among these genes, 463 presented increased expression, and 296 presented decreased expression, indicating that NLRP3 plays both activating and repressive roles in Th17 cells. Unsupervised hierarchical clustering and PCA revealed that *Nlrp3-*deficient Th17 cells adopted a transcriptomic profile closely resembling that of inflammatory Th17 cells (Fig. 2A, B).

On the basis of previous publications [16–19], we examined 31 genes associated with the inflammatory Th17 transcriptome. Seventeen of these genes were significantly upregulated in *Nlrp3*-deficient Th17 cells differentiated with TGF-β and IL-6 for 72 h (Fig. 2C). Time-course RNA-Seq analyses confirmed that this inflammatory signature progressively emerged at 24 h (Fig. 2D) and 72 h (Fig. 2E) of differentiation. Using RT‒PCR, we validated these findings over time courses of 24 h, 72 h and 7 days. At the 7-day time point, the cells were initially differentiated in vitro for 3 days. Each well was subsequently split, and the cells were restimulated for an additional 4 days without the addition of cytokines. The results confirmed that *Nlrp3-*deficient Th17 cells maintained a stable inflammatory profile over time (Fig. 2F). At the protein level, we validated the increased expression of the inflammatory cytokines IFNγ (Fig. 1a, b and Supplementary Fig. 2), TNFα, Granzyme B and GM-CSF (encoded by the *csf2* gene) in *Nlrp3*-deficient Th17 cells (Supplementary Fig. 5a, b). We further investigated the impact of deleting NLRP3 after Th17 cell differentiation. We generated *Nlrp3*^flox/flox^ × CD4^CreERT2^ mice, where Cre recombinase is induced by tamoxifen. We differentiated naïve CD4+ T cells into Th17 cells with TGF-β and IL-6 for 3 days, followed by 1 µM 4-hydroxytamoxifen treatment for 72 h. RT‒qPCR revealed that *Nlrp3* deletion after Th17 differentiation did not induce *Ifng* expression (Supplementary Fig. 5c). Finally, *Nlrp3* deficiency did not affect cytokine expression in pathogenic Th17 cells differentiated with TGF-β3 and IL-6 (Supplementary Fig. 5d–h).

Using B16F10 tumors in vivo, we validated the inflammatory profile of Th17 cells by isolating CD4+ CCR6+Foxp3- TILs. These Th17-enriched cells presented elevated expression of inflammatory genes in the CD4^*Nlrp3-/-*^ mice compared with the littermate controls (Fig. 2G).

The expression of *Il10* and *cMaf*, which are hallmarks of immunosuppressive Th17 cells [16, 20], was not altered by NLRP3 deficiency (Supplementary Fig. 6a–c). Additionally, the membrane expression of CD39 and CD73 ectonucleotidases, which mediate ATP-to-AMP conversion and confer immunosuppressive properties [21], was unaffected. There was no difference in the gene expression of *Entpd1* and *Nt5e*, which encode CD39 and CD73, respectively (Supplementary Fig. 6d, e), and no difference in their membrane expression (Supplementary Fig. 6f) was observed. Additionally, no difference was observed in ATP consumption (Supplementary Fig. 6g) or AMP production (Supplementary Fig. 6h). Inflammatory Th17 cells are characterized by increased glycolytic activity [22], but metabolic analysis revealed no shift in glycolysis in *Nlrp3*-deficient Th17 cells (Supplementary Fig. 6i–k).

Taken together, these findings demonstrate that *Nlrp3* deficiency induces a partial shift from an immunosuppressive phenotype to an inflammatory phenotype in Th17 cells.

### Th17 cells deficient in NLRP3 lose immunosuppressive functions

**Fig. 3:**
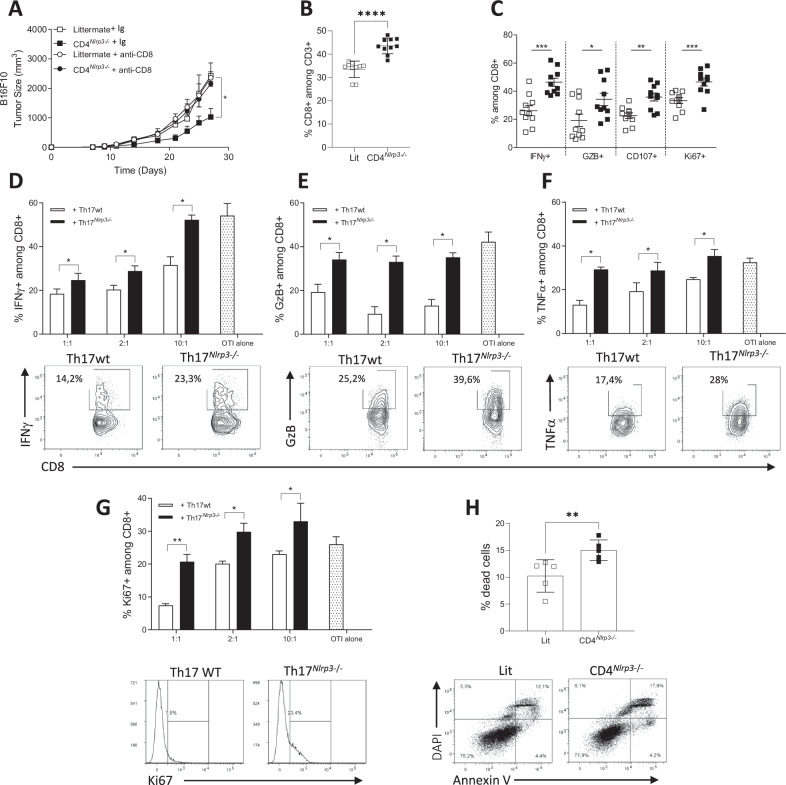
Th17 cells deficient in NLRP3 lose their immunosuppressive functions. **A**–**C** B16F10 tumor cells were injected subcutaneously into CD4^*Nlrp3*-/-^ mice (*n* = 10) and their littermate controls (Lit) with or without an anti-CD8a neutralizing antibody or a control Ig. **A** Tumor growth. **B**, **C** Analysis of CD8+ TILs by flow cytometry. **B** CD8+ frequency, **C** expression of IFNγ, granzyme B (GZB), and CD107a (CD107) and Ki67 staining. **D**–**G** Naïve CD4+ T cells were isolated from CD4^*Nlrp3*-/-^ mice and their littermate controls and differentiated into regulatory Th17 cells for 72 h (Th17wt, Th17^*Nlrp3-/-*^). CD8+ T lymphocytes were isolated from OTI mice and cultured alone or with Th17 cells at increasing CD8:Th17 ratios (1:1, 2:1, or 10:1). The production of IFNγ, **D** Granzyme B, **E** and TNFα, **F** and proliferation, **G** were evaluated via flow cytometry. **H** CD8+ T cells were isolated from B16F10 tumors from CD4^*Nlrp3-/-*^ mice and their littermate controls and cocultured with B16F10 cells for 24 h. The B16F10 mortality rate was assessed by flow cytometry of CD45- cells. The data shown are representative of 3 independent in vivo experiments or 5 independent in vitro experiments. Statistical significance was determined by 2-way ANOVA, Tukey’s multiple comparison test (**A**) and the Mann‒Whitney test (**B**–**H**). * <0.05, **<0.01, ***<0.005

We generated OTII^*Nlrp3-/-*^ mice with class II MHC specific for ovalbumin. Th17 cells differentiated from WT OTII or OTII^*Nlrp3-/-*^ mice in the presence of with TGF-β and IL-6 were not directly toxic to B16-OVA cells (Supplementary Fig. 7a). To explore whether Th17 cells indirectly affect tumor growth, we evaluated immune cell dependencies. Depletion of NK cells or macrophages with an NK-depleting mAb or clodronate liposomes did not significantly affect tumor growth in CD4^*Nlrp3-/-*^ mice (Supplementary Fig. 7b, c). In contrast, the anti-CD8 depleting antibody abrogated the delay in tumor growth observed in the CD4^*Nlrp3-/-*^ mice (Fig. 3A and Supplementary Fig. 7d, e). Flow cytometry analysis revealed the accumulation of CD8+ T cells in tumors from CD4^*Nlrp3-/-*^ mice (Fig. 3B and Supplementary Fig. 7f). These cells produced more IFNγ, Granzyme B, and CD107a and exhibited increased proliferation (Fig. 3C) compared with littermate controls, with no effect on checkpoint inhibitor receptor expression (not shown).

To investigate the immunosuppressive activity of Th17 cells in vitro, CD8+ cells from OTI mice (expressing class I MHC specific for ovalbumin) were stimulated with the SIINFEKL peptide and cocultured with Th17 cells differentiated from either OTII or OTII^*Nlrp3-/-*^ naïve CD4+ T cells in the presence of TGF-β and IL-6. While WT Th17 cells suppressed IFNγ, Granzyme B, and TNFα production in CD8+ T cells, Th17 cells deficient in *Nlrp3* did not (Fig. 3D–F), nor did they inhibit CD8+ cell proliferation (Fig. 3G). Ex vivo coculture of CD8+ TILs from CD4^*Nlrp3-/-*^ mice with B16F10 tumors and their littermate controls for 24 h confirmed that CD8+ cells from CD4^*Nlrp3-/-*^ mice exhibited greater cytotoxic activity than cells sorted from littermate controls did (Fig. 3H).

Taken together, these results highlight that NLRP3 in Th17 cells limits CD8+ cell antitumor activity.

### Noncanonical functions of NLRP3 are involved in Th17 cells

The NLRP3 inflammasome has been implicated in CD4+ T-cell differentiation [23, 24]. However, we previously demonstrated that the NLRP3 inflammasome is not involved in our experimental conditions and does not modulate cytokine gene expression in Th17 cells (Supplementary Fig. 3l–m). *Ifnγ* can be regulated by transcription factors such as T-bet (encoded *by the Tbx21* gene), IRF1, or Eomes. We therefore analyzed their expression over time in *Nlrp3*-deficient Th17 cells. Surprisingly, increased *Ifnγ* expression was induced earlier than T-bet, IRF1, or Eomes expression, suggesting that these transcription factors are not involved in *Ifnγ* regulation in this context (Supplementary Fig. 8a–d). *Ifnγ* expression can also be induced through the STAT1 pathway, but STAT1 phosphorylation was identical in WT and *Nlrp3*-deficient cells (Supplementary Fig. 8e, f).

**Fig. 4:**
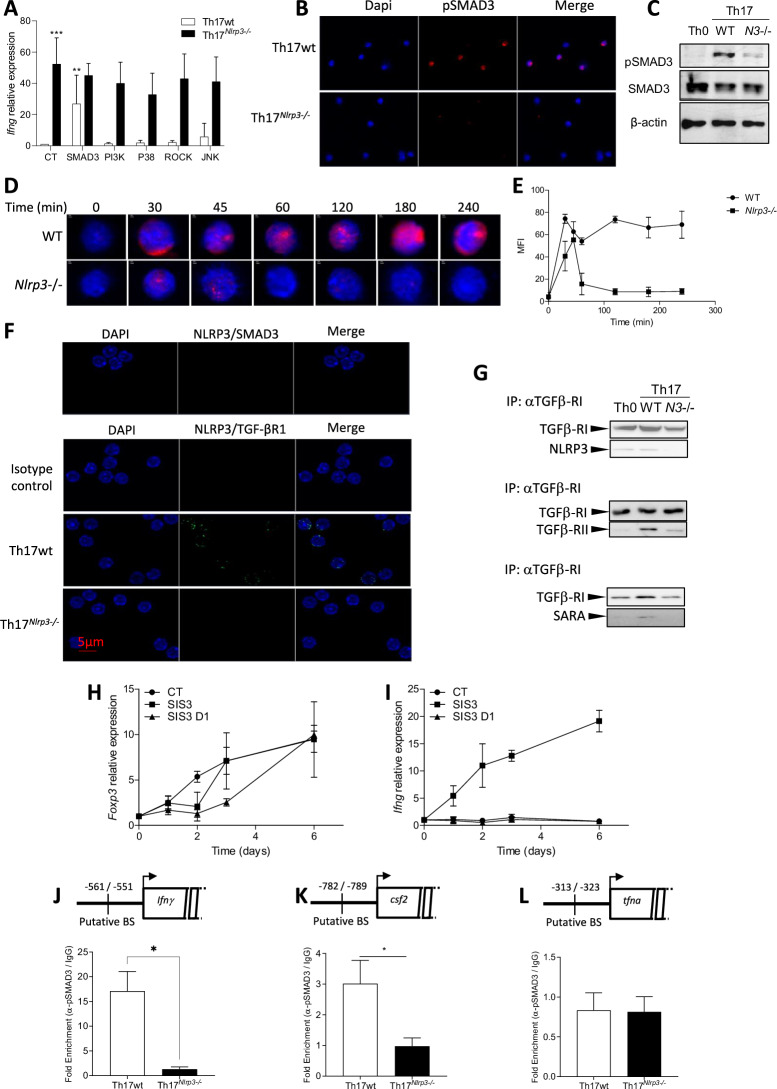
NLRP3 inflammasome-independent functions are involved in Th17 cells. **A** *Ifnγ* mRNA levels in *Nlrp3*-deficient Th17 cells (Th17^*Nlrp3*-/-^) and their controls (Th17wt) after 24 h of in vitro polarization, treated or not (NT) with pharmacological inhibitors of SMAD3 (SIS3), PI3K (LY294002), P38 (SB203580), ROCK (Y27632) or JNK (JNK Inhibitor II). **B** Immunofluorescence staining of pSMAD3 in Th17 cells differentiated in vitro for 24 h from naïve CD4^*Nlrp3*-/-^ deficient mice (Th17^*Nlrp3*-/-^) and their littermate controls (Th17wt). **C** Cells were differentiated as described previously (Th0, Th17WT, and Th17^*Nlrp3*-/-^) for 24 h, lysed, and analyzed via Western blotting with the indicated antibodies. Immunofluorescence staining (**D**) and quantification (**E**) of pSMAD3 in Th17WT or Th17^*Nlrp3*-/-^ cells during short-term kinetics. **F** Proximity of NLRP3 and pSMAD3 (upper panel) and NLRP3 and TGF-βR1 (lower panel) detected by proximity ligation assay in Th17 cells differentiated for 24 h in vitro from naïve CD4^*Nlrp3*-/-^ deficient mice (Th17^*Nlrp3*-/-^) and their littermate controls (Th17wt). **G** Cells were differentiated as described in (**C**), harvested and lysed, followed by immunoprecipitation with an anti-TGF-β-RI antibody and Western blot analysis with the indicated antibodies. **H** Relative *Foxp3* expression in Th17 cells treated with or without the SMAD3 inhibitor SIS3 either from the start of differentiation or after 1 day of differentiation. **I** *Ifnγ* relative expression under the same conditions as in (**H**). pSMAD3 enrichment at putative pSMAD3 binding sites on the *Ifnγ* (**J**), *csf2* (**K**) and *Tnfα* (**L**) promoters by chromatin immunoprecipitation (ChIP) assay in Th17 cells differentiated for 1 h in vitro from naïve CD4^*Nlrp3*-/-^ deficient mice (Th17^*Nlrp3*-/-^) and their littermate controls (Th17wt). The data shown are representative of 4–5 independent experiments. Statistical significance was determined by the Mann‒Whitney test. * <0.05, **<0.01, ***<0.005

Th17 cells differentiate in the presence of IL-6 and TGF-β, and TGF-β can inhibit the effector functions of immune cells and limit the production of inflammatory cytokines, such as IFNγ. To determine whether “NLRP3 signaling” intersects with the TGF-β pathway, we used pharmacological inhibitors targeting various signaling pathways downstream of the TGF-β receptor. The application of an SMAD3 inhibitor during Th17 differentiation increased *Ifnγ*, *tnfα*, and *Gzmb* expression in WT Th17 cells but had no effect on Th17 cells deficient in *Nlrp3* (Fig. 4A and Supplementary Fig. 8g, h). TGF-β receptor activation during Th17 differentiation induces SMAD3 phosphorylation, as detected by immunofluorescence, Western blot, and cytometry in WT Th17 cells. However, SMAD3 phosphorylation was largely abolished in *Nlrp3*-deficient Th17 cells (Fig. 4B, C and Supplementary Fig. 8i). A time course study revealed that SMAD3 phosphorylation is initiated very early in WT Th17 cells (within 30 min) and remains. *Nlrp3*-deficient Th17 cells exhibited only weak phosphorylation signals early (30 and 45 min after the start of differentiation) and lost this signal by 60 min (Fig. 4D, E). In contrast, STAT3 phosphorylation was unaffected by *Nlrp3* deficiency (Supplementary Fig. 8j). Furthermore, neither NLRP3 inflammasome activation nor inhibition affected SMAD3 phosphorylation (Supplementary Fig. 8k). However, total SMAD3 levels remained unchanged (Fig. 4C and Supplementary Fig. 8k). Using a Proximity Ligation Assay (PLA), no interaction was detected between NLRP3 and SMAD3 or SMAD2 (Fig. 4F upper panel). However, an interaction between NLRP3 and TGF-β receptor I was detected (Fig. 4F, lower panel). Coimmunoprecipitation experiments after 24 h of differentiation confirmed the interaction between NLRP3 and TGF-β receptor I, even without TGF-β stimulation. These experiments also revealed reduced formation of the TGF-β receptor complex, which involves the SARA protein, in *Nlrp3*-deficient Th17 cells during their differentiation (Fig. 4G).

The TGF-β pathway, via SMAD3 activation, induces *Foxp3* expression. Our data revealed a progressive increase in *Foxp3* expression over time in WT Th17 cells (Supplementary Fig. 8l). Neither activation nor inhibition of the NLRP3 inflammasome influenced *Foxp3* expression (Supplementary Fig. 8m). FOXP3 has been reported to inhibit *Ifnγ* transcription, notably through a complex with RUNX1 [25]. To evaluate the relationships among SMAD3, FOXP3, and *Ifnγ* expression, we treated Th17 cells with the SMAD3 inhibitor SIS3. When SIS3 was added at the start of differentiation, *Foxp3* induction was blocked during the first two days, followed by delayed induction (Fig. 4H), whereas *Ifnγ* expression was induced very early (Fig. 4I). In contrast, after 24 h of differentiation, SIS3 treatment minimally affected *foxp3* expression before day 5 and did not induce *Ifnγ* expression (Fig. 4H, I). These results suggest that *Ifnγ* inhibition in Th17 cells requires early SMAD3 activation during differentiation in the presence of TGF-β and IL-6. To confirm whether SMAD3 is responsible for inhibiting *Ifnγ* transcription, we analyzed the *Ifnγ* promoter with Matinspector and identified a putative pSMAD3 binding site at -551 to -561. ChIP analysis revealed that pSMAD3 bound to the *Ifnγ* promoter in WT Th17 cells but not in *Nlrp3*-deficient Th17 cells (Fig. 4J). We previously reported that Granzyme B, TNFα, and GM-CSF were induced in *Nlrp3*-deficient Th17 cells (Supplementary Fig. 5a, b). Analysis of their promoters with Matinspector identified putative pSMAD3 binding sites: −782 to −789 for *Csf2* and −313 to −323 for *Tfnα,* but no putative binding site in the *Gzmb* promoter. ChIP experiments revealed that pSMAD3 bound to the *Csf2* promoter (Fig. 4K) in WT Th17 cells but not at *Tnfα* (Fig. 4L). Granzyme B, TNFα, and GM-CSF can also be induced by IFNγ. We tested the effect of an IFNγ blocking antibody during Th17 differentiation and found that IFNγ regulated only *Granzyme B* expression (Supplementary Fig. 8n–s).

Together, these data indicate that NLRP3 participates in the differentiation of regulatory Th17 cells by stabilizing SMAD3 phosphorylation. Once activated, pSMAD3 inhibits *Ifng* and *Csf2* expression.

### NLRP3 deficiency inhibits the conversion of Th17 cells into Treg cells

**Fig. 5:**
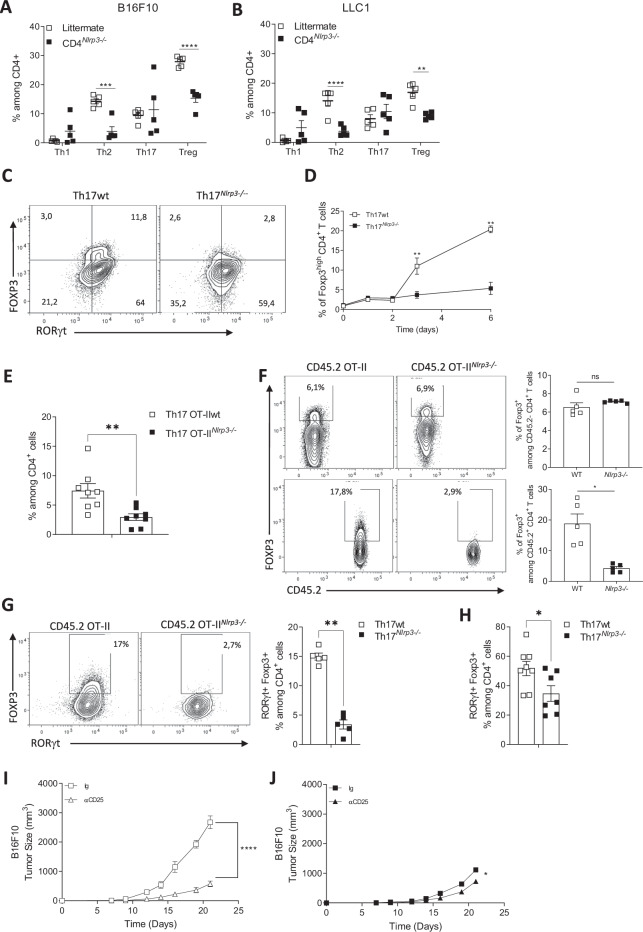
NLRP3 deficiency inhibits the transdifferentiation of Th17 cells into Treg cells. Proportion of CD4+ T-cell subsets (Th1, Th2, Th17, and Treg) in subcutaneous B16F10 (**A**) and LLC1 (**B**) tumors from CD4^*Nlrp3*-/-^ mice and their littermate controls (*n* = 5). Naïve CD4+ T cells isolated from CD4^*Nlrp3*-/-^ mice and their littermate controls were differentiated in vitro into Th17 cells. The expression of RORγt and FOXP3 was detected by cytometry over a time course on days 0, 1, 2, 3 (**C**) and 6 (**D**). **E**–**H** Th17 cells were differentiated from naïve CD4+ T cells isolated from CD45.2 OTII WT or *Nlrp3*-deficient mice. These cells were transferred into WT CD45.1 mice (**E**‒**G**) or into CD4^*Rorc*-/-^ mice bearing B16‒OVA lung tumors (**H**). Forty-eight hours later, the frequencies of Treg cells present in the lungs were analyzed by cytometry among CD4+ T cells (**E**) (*n* = 8), among CD45.2+ and CD45.2− cells (*n* = 5) (**F**), and among RORγt + cells (**G**, **H**) (*n* = 5 in **G** and *n* = 8 in **H**). Growth of subcutaneously injected B16F10 melanoma tumors in CD4^*Nlrp3*-/-^ mice (**I**) and their littermate controls (**J**) not treated with an anti-CD25 blocking antibody (*n* = 5). The data are representative of 3 independent experiments. Statistical significance was determined by the Mann‒Whitney test (**A‒H**), 2-way ANOVA and Tukey’s multiple comparison test (**I‒J**). * <0.05, **<0.01, ***<0.005

As expected, flow cytometry analysis of CD4+ TILs (Supplementary Fig. 9a) in both B16F10 (Fig. 5A) and LLC1 tumors (Fig. 5B) revealed a reduced frequency of Th2 cells. Surprisingly, Tregs were also reduced in CD4^*Nlrp3-/-*^ mice compared with their littermate controls. This result contrasts with our in vitro data, which indicate that *Nlrp3* deficiency does not affect Treg differentiation (Supplementary Fig. 2 and Supplementary Fig. 9b), as well as with the in vivo findings showing that *Nlrp3* deficiency does not affect the Treg proportion in the thymus or secondary lymphoid organs (Supplementary Fig. 9c, d). Furthermore, we observed no difference in the activation of Tregs in tumors, as assessed by the membrane markers CCR4, CCR8, CD137, CTLA-4, LAG-3, PD-1, and TIGIT, or in their proliferation, as indicated by Ki67 staining (Supplementary Fig. 9e). Finally, their recruitment, as assessed via an in vivo adoptive transfer experiment, was also unaffected (Supplementary Fig. 9f).

We hypothesized that NLRP3 influences the capacity of Th17 cells to transdifferentiate into Tregs. Naïve CD4 + T cells from CD4^*Nlrp3-/-*^ mice and controls were differentiated into Th17 cells in vitro in the presence of TGF-β and IL-6. A subset of RORγt + Th17 cells expressing FOXP3 was observed in control mice on day 3, but this subset was absent in Th17 cells deficient in *Nlrp3* (Fig. 5C). The number of WT Th17 cells expressing FOXP3, but not Th17 cells deficient in *Nlrp3* increased over time (Fig. 5D and Supplementary Fig. 8l). To assess whether this difference is observable in vivo, we differentiated naïve CD4+ T cells from CD45.2 OTII or CD45.2 OTII mice deficient in *Nlrp3* into Th17 cells in vitro in the presence of TGF-β and IL-6. These cells were transferred into congenic WT mice (expressing the CD45.1 allele) bearing B16-OVA tumors. After 48 h, lung CD4+ infiltrates were analyzed, revealing a greater proportion of FOXP3+ cells in mice receiving WT Th17 cells than in those receiving *Nlrp3*-deficient Th17 cells (Fig. 5E). Importantly, no differences in Th1 or Th17 frequencies, as identified by their surface markers, were detected (Supplementary Fig. 10a). Analysis of TILs revealed that FOXP3+ CD4+ T cells from CD45.1 host mice were unaffected by Th17 adoptive transfer (Fig. 5F). However, the proportion of FOXP3+ cells among transferred CD45.2 cells was greater in the mice receiving WT Th17 cells than in those receiving *Nlrp3*-deficient Th17 cells (Fig. 5F). Similarly, cells simultaneously expressing FOXP3 and RORγt were more likely to differentiate into WT Th17 cells than into *Nlrp3*-deficient Th17 cells (Fig. 5G). These findings were replicated in CD4^*Rorc-/-*^ mice, which lack endogenous Th17 cells, in adoptive transfer experiments with WT OTII Th17 or OTII Th17^*Nlrp3-/-*^ cells (Fig. 5H). Finally, we used an anti-CD25 blocking antibody to target Tregs in vivo during B16F10 tumor growth. Tumor growth was significantly slowed in control mice treated with the antibody (Fig. 5I), whereas CD25 depletion only modestly affected tumor growth in CD4^*Nlrp3-/-*^ mice (Fig. 5J).

Taken together, these data suggest that NLRP3 promotes *Foxp3* expression in Th17 cells and facilitates their conversion into Tregs, thereby promoting tumor growth.

### Targeting NLRP3 in Th17 cells has therapeutic value

**Fig. 6:**
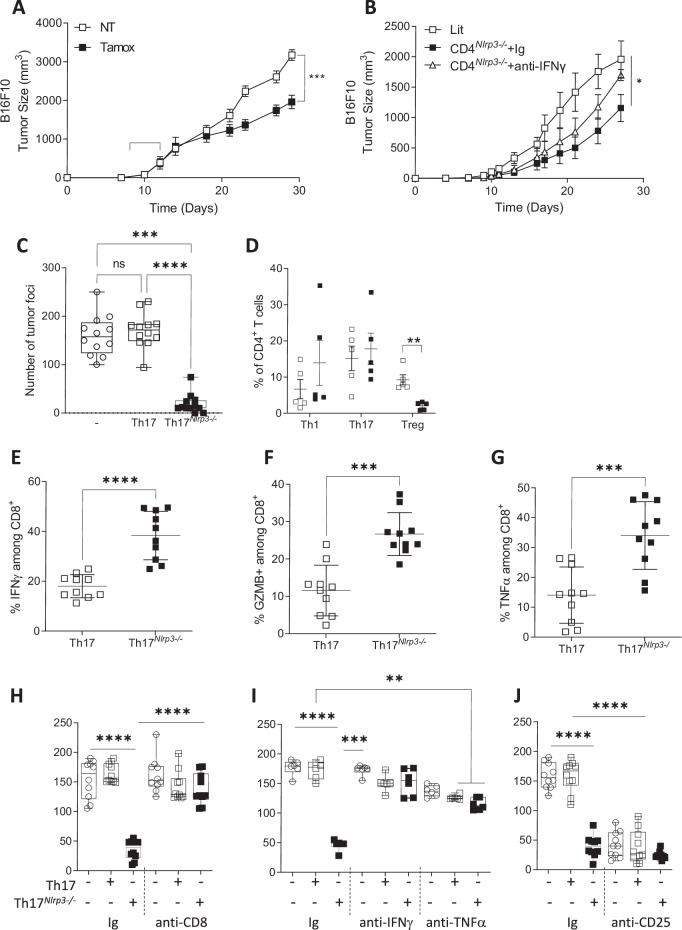
Thus, targeting NLRP3 in Th17 cells has therapeutic value. **A** Tumor growth of subcutaneously injected B16F10 melanoma in *Nlrp3*^*flox/flox*^ × CD4^CreERT2^ mice treated or not treated with tamoxifen (brackets indicate the days of tamoxifen administration) (*n* = 5). **B** Tumor growth of CD4^*Nlrp3*-/-^ mice and their littermate controls subcutaneously injected with B16F10 melanoma cells. CD4^*Nlrp3*-/-^ mice were treated twice a week with an anti-IFNγ blocking antibody or a control Ig (*n* = 5). **C** B16-OVA lung tumor foci 13 days after intravenous injection of OTII Th17 (Th17) cells or OTII *Nlrp3*-deficient Th17 cells (Th17^*Nlrp3-/-*^) (*n* = 12). **D** Proportion of CD4+ T-cell subsets (Th1, Th17, and Treg) in the lungs of B16-OVA tumor-bearing mice 13 days after intravenous injection of OTII Th17 (white squares) or OTII *Nlrp3*-deficient Th17 cells (black squares) (*n* = 5). **E**–**G** (*n* = 12), Analysis of CD8+ cells from the lungs of C mice via flow cytometry. **D** IFNγ production, **E** granzyme B production (**F**), and TNFα production. **H**–**J** B16-OVA lung tumor foci in mice treated with OTII Th17 (Th17) or OTII *Nlrp3*-deficient Th17 cells (Th17^*Nlrp3-/-*^) with or without (**H**, *n* = 10) anti-CD8 blocking antibody injected twice a week, (**I**, *n* = 6) anti-IFNγ or anti-TNFα blocking antibodies injected twice a week or (**J**, *n* = 10) anti-CD25 blocking antibody injected twice a week. The data are representative of 3 independent experiments. Statistical significance was determined by Tukey’s multiple comparison test (**A**, **B**) and 2-way ANOVA (**C**–**J**). * <0.05, **<0.01, ***<0.005, ****0.0001

To explore the therapeutic potential of targeting NLRP3, we used the *Nlrp3*^flox/flox^ × CD4^CreERT2^ mice that we previously generated. Tamoxifen treatment, initiated when tumors were palpable, slowed B16F10 tumor growth (Fig. 6A). Since our data indicate that *Nlrp3*-deficient Th17 cells produce increased levels of IFNγ and TNFα, we studied the impact of neutralizing these cytokines in vivo. TNFα-neutralizing antibodies had no effect, whereas IFNγ neutralization reduced the inhibition of tumor growth (Fig. 6B and Supplementary Fig. 10b). To evaluate the therapeutic efficacy of *Nlrp3*-deficient Th17 cells, we conducted adoptive transfer experiments. In line with ethical considerations, we used a pulmonary B16-OVA tumor model, which requires fewer CD4+ cells (and therefore fewer mice for cell production) than subcutaneous tumor models do to achieve a therapeutic effect. We observed that adoptive transfer of Th17 cells differentiated from OTII^*Nlrp3-/-*^ mice into WT mice reduced the number of B16-OVA lung tumor foci (Fig. 6C). Analysis of tumor infiltrates revealed more Tregs in mice receiving WT Th17 cells than in those receiving *Nlrp3*-deficient Th17 cells (Fig. 6D). Additionally, CD8+ cells in the *Nlrp3*-deficient Th17 cells of the mice presented increased IFNγ (Fig. 6E), Granzyme B (Fig. 6F) and TNFα (Fig. 6G) levels. The anti-CD8 depleting antibody completely abrogated the antitumor effect of *Nlrp3*-deficient OTII Th17 cells, underscoring the essential role of CD8+ cells in this therapeutic context (Fig. 6H). Since our data indicate that  Nlrp3-deficient Th17 cells produce increased levels of IFNγ and TNFα, we investigated the impact of neutralizing TNFα and IFNγ by using neutralizing antibodies administered according to the same protocol as anti-CD8. TNFα inhibition had an intermediate effect. In contrast, IFNγ inhibition completely counteracted the antitumor effects of adoptive transfer of *Nlrp3*-deficient OTII Th17 cells (Fig. 6I). This finding was corroborated in *Ifnγ* receptor-deficient mice, where adoptive transfer of *Nlrp3*-deficient OTII Th17 cells failed to elicit antitumor effects (Supplementary Fig. 10c). The anti-CD25 antibody reduced the number of lung tumor foci in mice receiving WT Th17 cells but did not enhance the antitumor effect of *Nlrp3*-deficient Th17 cells (Fig. 6J). CD8+ T cells in these tumors were increased by anti-CD25 therapy in the littermate control mice, but this effect was not detected in the CD4^*Nlrp3-/-*^ mice (Supplementary Fig. 10d).

Taken together, these data highlight the potential of targeting NLRP3 functions in CD4+ T cells as a therapeutic strategy.

## Discussion

In this study, we demonstrated that NLRP3 is required for the transdifferentiation of Th17 cells into Tregs. Notably, NLRP3 performs noncanonical functions in Th17 cells, interacting with the TGF-β receptor and participating in the TGF-β-SMAD3 signaling pathway. Consequently, NLRP3 inhibits the production of inflammatory cytokines, promoting an immunosuppressive phenotype of Th17 cells while facilitating their conversion into Tregs. Using transgenic mouse models, we observed that targeting NLRP3 in CD4+ T cells, and by extension, in Th17 cells, reduced the immunosuppressive impact on CD8+ cells and enhanced their antitumor functions.

NLRP3 is best known for its role in the inflammasome, which forms in myeloid cells after detection of danger signals. Consequently, most cancer-related studies on NLRP3 have focused on its inflammasome function, with conflicting findings. For example, mice deficient in *Nlrp3* or other inflammasome components are more susceptible to colon tumors following AzOxyMethane/Dextran Sodium Sulfate treatment, suggesting a protective role of the NLRP3 inflammasome in colorectal cancers [26]. This protective effect may arise from increased IL-1β, which facilitates microbiota remodeling and induces Tregs, compensating for deleterious inflammation [27]. In mice, *Nlrp3* deficiency has been linked to fewer lung metastases in an orthotopic tumor model [28]. Additionally, NLRP3 has been implicated in promoting gastric and cutaneous tumor development [29]. High expression of *Nlrp3* is correlated with poor survival in patients with breast cancer, likely through its role in inducing Epithelial‒Mesenchymal Transition (EMT) [30], as well as in patients with colorectal cancer [31]. The lack of significant differences in tumor growth between fully *Nlrp3*-deficient mice and CD4^*Nlrp3*fl/fl^ mice that we observed suggests a predominant role of NLRP3 in CD4+ T cells during tumor progression. Notably, the absence of NLRP3 exclusively in CD4+ T cells is sufficient to replicate the slowed tumor growth observed in mice completely deficient in *Nlrp3*. These findings indicate that the deleterious role of NLRP3 in other cell types (such as myeloid cells), particularly through IL-1β secretion, may be compensated for by the blockade of NLRP3 in CD4+ T cells.

The role of NLRP3 in CD4+ T cells is relatively understudied. In 2015, our team was the first to demonstrate that NLRP3 expression in CD4+ T cells is driven by an IL-2-dependent pathway. We established its function as a transcription factor in Th2 cells, contributing to their protumor properties [11]. In this new study, we extended our findings to show that NLRP3 also impacts Th17 cells. The altered phenotype of *Nlrp3*-deficient Th17 cells is independent of the classical inflammasome function of NLRP3. Specifically, inhibiting Caspase-1 (which is involved in the classical inflammasome) or Caspase-8 (which is involved in the inducible inflammasome in Th17 cells [12]) does not result in an increase in IFNγ expression in Th17 cells. In Martin et al.’s study [12], the absence of ASC or NLRP3 in Th17 cells reduced both IFNγ production and cell proliferation, supporting our observations that NLRP3 functions independently of the inflammasome in Th17 cells.

The phenotypic changes in *Nlrp3-*deficient Th17 cells are associated with a reduction in tumor growth, in which Th17 cells are implicated. Importantly, our findings diverge from those of Park SH et al., as we did not observe any direct role of NLRP3 in Treg differentiation [32]. Under our experimental conditions, we found no evidence of inflammasome activation in Th17 cells, as reflected by the absence of cytokine production (IL-1β and IL-18) and the lack of effect of inflammasome inhibitors. Additionally, CD4+ T cells presented minimal to no expression of *Caspase-1* or *Tlr4*. While there may be inflammasome-dependent and inflammasome-independent roles of NLRP3 in Th17 cells, our experimental conditions suggest that the regulation of the inflammatory/regulatory profile of Th17 cells does not rely on inflammasome activity. In a study by Doitsh et al. [10], the authors focused on human CD4+ T lymphocytes in general, without specifically examining Th17 cells. Additionally, their inflammasome activation conditions are specific, as they are induced by HIV. Our data indicate that typical inflammasome activation conditions used in macrophages, such as LPS + ATP, fail to activate the inflammasome in Th17 cells, which is consistent with the low or negligible *Tlr4* expression we observed. Chao et al. [14] demonstrated that TCR activation in human CD4+ T cells induces the expression of *il-1a*, *Casp-8, calpain*, etc. We observed similar gene induction in our experiments. However, when we used inhibitors of Caspase-8, inflammasome formation, and IL-1RA (the receptor for IL-1α and IL-1β), we did not detect an increase in *Ifng* expression in Th17 cells, contrary to the effect observed with NLRP3 deficiency. These findings underscore an inflammasome-independent function of NLRP3 in Th17 cells.

Th17 cells are identified by their production of IL-17 and expression of the transcription factor RORγt [33, 34]. Their role in cancer is a subject of debate and varies with the tumor site and the local cytokine milieu influencing cell differentiation. In the presence of TGF-β, Th17 cells exhibit an immunosuppressive phenotype characterized by the expression of *cMaf, Il-10*, and the ectonucleotidases CD39 and CD73 and the release of adenosine [21]. Conversely, Th17 cells differentiate without TGF-β, express T-bet and produce IFNγ, adopting an inflammatory phenotype associated with Experimental Autoimmune Encephalomyelitis [20]. In our study, Nlrp3-deficient Th17 cells differentiated in vitro in the presence of TGF-β displayed a transcriptome resembling that of inflammatory Th17 cells described in the literature [17–19]. This phenotype persisted in vivo, within the tumor environment, and appeared to shift the tumor toward a less immunosuppressive environment.

Adoptive transfer experiments have demonstrated that Th17 cells possess antitumor potential mediated by IFNγ rather than by IL-17A [35]. This effect involves an inflammatory response that recruits DCs, NK cells [36], and CD8 + T cells, increasing their proliferation and cytotoxicity [37]. However, Th17 cells can also suppress antitumor immunity [38] through mechanisms such as adenosine release under TGF-β conditions [21] or conversion into Tregs [8]. In our study, *Nlrp3-*deficient Th17 cells lost their immunosuppressive properties, enhancing CD8+ T-cell activity both in vitro and in vivo. Although these cells retain their capacity for ectonucleotidase expression and adenosine production, their immunosuppressive activity is counterbalanced by the release of inflammatory cytokines. Specifically, *Nlrp3-*deficient Th17 cells produce IFNγ, directly stimulating CD8+ cells, as does GM-CSF, which activates CD8+ cells through dendritic cell activation [36, 37]. Additionally, TNFα produced by Th17 cells facilitates the activation, proliferation and recruitment of CD8+ cells via the TNFR2 receptor [39]. While we did not observe differences in other inflammatory cytokine-producing cells or changes in IFNγ production by Th1 cells, it is likely that Th17 cells influence the cytokine production of other immune cells. For example, macrophage differentiation and polarization toward the M1 phenotype, characterized by inflammatory cytokine production, are promoted by IFNγ [40]. Similarly, TNFα is known to activate macrophages [41] and support NK cell proliferation [40–42].

In mice, IL-6 and TGF-β induce immunosuppressive Th17 cells [21, 43]. IL-6 activates the STAT3 pathway, which is critical for IL-17 production [1], whereas TGF-β triggers various signaling cascades, including the SMAD, TRAF6/TAK1, and PI3K/Akt, MAPK, P38, ERK, and JNK pathways, as well as Rho-like GTPases [44]. SMAD3 phosphorylation by the TGF-β receptor facilitates SMAD4 nuclear translocation, acting as a transcription factor regulating gene expression. In Th17 cells, pSMAD3 suppresses T-bet and STAT4, reducing IFNγ expression [45, 46]. The FOXP3 transcription factor, which is induced via TGF-β-SMAD3 signaling, also inhibits IFNγ expression [25]. In our study, *Nlrp3-*deficient Th17 cells produced IFNγ, GM-CSF, and TNFα independently of the transcription factors previously described to regulate their expression (T-bet, Eomes, IRF1, and STAT1). We observed that NLRP3 interacts with the TGF-β receptor, stabilizing SMAD3 phosphorylation to inhibit the expression of *Ifnγ* by binding to its promoter region. Without NLRP3, SMAD3 phosphorylation is transient, leading to very early expression of *Ifnγ* during differentiation.

Surprisingly, no effect was observed on Treg cells, even though their differentiation largely depends on the TGF-β pathway. However, Th17 cell differentiation relies on signals induced by both TGF-β and IL-6, and these pathways influence each other. In the absence of IL-6, TGF-β alone increases *Foxp3* expression, thereby promoting Treg differentiation. IL-6 blocks this effect of TGF-β by inhibiting *Foxp3* via STAT3 [47]. Conversely, synergy between IL-6 and TGF-β stimulates IL-21 production as well as the expression of the IL-23 receptor [48]. Furthermore, although it has not yet been formally demonstrated, a link between IL-6 and iSMADs (inhibitory SMADs, particularly SMAD7) appears to play a role in the regulation of the TGF-β pathway. Indeed, IL-6 activates the JAK/STAT3 pathway, and STAT3 can induce the expression of *Smad7* [49, 50], an inhibitor of TGF-β signaling that limits the phosphorylation and activation of SMAD2/3. Additionally, since Tregs produce very little IL-2, the expression level of NLRP3 in these cells is very low and appears to play only a minor role.

While TGF-β through SMAD3 is linked to *Il10* expression in Tregs [51], its role in Th17 cells is debated. McGeachy et al. reported that *Il10* induction, concurrent with *Rorc* induction, requires combined stimulation with both TGF-β and IL-6 [20]. Other studies suggest that FOXP3 induces *Il10*, whereas RORγt inhibits it [52]. Transcription factors such as BLIMP1 [53] or ASCL2 [54] also regulate *Il10* in Th17 cells, but many studies agree that c-MAF is a dominant inducer of this expression [55–62]. Activin A regulates *Il10* expression via SMAD3, with a proposed link between STAT3 and SMAD3, which we did not observe. In our experiments, neither *cMaf* nor *Il10* expression was altered in *Nlrp3*-deficient Th17 cells. Our results are consistent with some of the literature indicating that *cMaf* is induced by the STAT3 pathway in Th17 cells [63]. Indeed, we found that STAT3 phosphorylation was unaffected. Thus, our results indicate that NLRP3 has a partial effect on the Th17 profile, and further research is necessary to explore the respective effects of NLRP3 and cMaf in more detail. Previous studies linked NLRP3 to the TGF-β/SMAD3 pathway. A study showed that pSMAD3 is abolished in *Nlrp3-* and *Asc*-deficient mice, resulting in a decrease in TGF-β-induced EMT in cancerous kidney cells via an inflammasome-independent mechanism [64]. In our study, the results were different in *Nlrp3-* and *Asc*-deficient cells. In cardiac fibroblasts, *Nlrp3*-deficient mice presented reduced pSMAD3 due to ROS production [65], a mechanism not observed in our study (not shown).

Th17 cells are known for their plasticity, allowing them to transdifferentiate into Th1-like or Treg-like cells. Under the influence of IL-12 or IL-23, these cells can shift to a Th1-like phenotype [66, 67] as well as under the effects of inflammatory responses [68]. Exposure to TGF-β and/or prostaglandin E2 drives them toward a Treg profile [8]. Furthermore, the use of fate-mapping mice allowed the detection of Tregs expressing low levels of Foxp3 in the intestines of these mice. These cells are thought to have deviated from a Th17 fate [69]. In our study, we observed a reduction in Th2 cells as expected [11] but also a significant decrease in Tregs within CD4+ TILs from CD4^*Nlrp3-/-*^ mice compared with those from control mice. Although NRLP3 does not directly affect Treg differentiation, we focused on Th17 cells and discovered that the absence of *Nlrp3* impaired their ability to acquire *Foxp3* expression and transdifferentiate into Tregs both in vitro and in vivo. Th17-to-Treg cells involve various factors, including a switch in glucose metabolism [22] and the inhibition of fatty acid oxidation, which supports Treg development [70]. Our results highlight the pivotal role of NLRP3 in the TGF-β pathway, which is crucial for enabling this transdifferentiation.

Our findings confirm that Th17 cells are a significant source of Tregs in the TME. The identification of a new transdifferentiation pathway opens up potential therapeutic avenues to target Tregs and alleviate immunosuppression. However, we acknowledge certain limitations in our study. The adoptive transfer of in vitro-differentiated Th17 cells without the use of Rorc reporter mice may introduce bias since our differentiation protocol does not achieve 100% efficiency. A transcriptomic analysis also demonstrated the impact of the differentiation method [71]. Moreover, rapid changes have been observed both in vitro and in vivo after adoptive transfer, particularly at the epigenetic level, affecting the expression of transcription factor genes as well as cytokine genes [72, 73]. These limitations could influence the interpretability of our data. Nevertheless, our results consistently demonstrated that WT Th17 cells express *Foxp3* both in vitro and in vivo, in contrast to their *Nlrp3*-deficient counterparts. This acquisition of *Foxp3* expression underscores the functional importance of NLRP3 in modulating Th17 cell differentiation and suggests a preferential pathway for the induction of *Foxp3* in the presence of functional NLRP3. Rather than eliminating Tregs as a whole, selectively disrupting Th17 cell transdifferentiation via NLRP3 inhibition in CD4+ T cells could reduce Tregs while enhancing IFNγ production by Th17 cells. In support of this hypothesis, previous research has indicated that NLRP3 may limit T-cell-mediated antitumor immunity [74, 75]. A recent study further demonstrated that pharmacological inhibition of NLRP3 suppresses MDSC recruitment and enhances the efficacy of anti-PD-1 immunotherapy [76]. While most therapeutic strategies have focused on its inflammasome-dependent functions, our results highlight its noncanonical role in regulating Th17 cells, which remain unaffected by inflammasome inhibitors. In the context of adoptive T-cell therapy, preclinical data have shown that CD4+ T-cell transfer can mediate tumor rejection [77]. Our study suggested that inhibiting NLRP3 could enhance the efficacy of such approaches, as *Nlrp3*-deficient Th17 cells demonstrated a robust antitumor effect associated with an increase in CD8+ T-cell activity.

In summary, our study revealed that, through its interaction with the TGF-β receptor, NLRP3 facilitates SMAD3 phosphorylation, thereby suppressing inflammatory cytokine production in Th17 cells and enabling their transdifferentiation into Tregs. These findings identify NLRP3 as a crucial regulator of Th17 plasticity and a potential target for therapeutic strategies aimed at reducing Tregs in tumors. By targeting the noncanonical functions of NLRP3 in CD4+ T cells, enhancing antitumor immunity while maintaining a balanced immune response may be possible.

## Materials and methods

### Mice

C57BL/6J mice were purchased from Charles River Laboratories (Saint Germain sur l’Arbresle, France). *Nlrp3*^flox/flox^CD4^Cre^ mice and their littermate controls *Nlrp3*^flox/flox^CD4^wt^ mice, *Nlrp3*^flox/flox^CD4^creERT2^ mice and their littermate controls *Nlrp3*^flox/flox^CD4^wt^, OT-II, OT-II and *Nlrp3*-/-, were bred at the CDTA (Cryopreservation, Distribution, Typage et Archivage animal; Orléans, France), such as *Asc*-/-, *Caspase1*-/-, *Il1receptor type I*-/- and *Il18r*-/-, *IfnγR*-/- mice and *Rorc*^fl/fl^CD4cre (kindly provided by Pr Ryffel - CNRS, Orléans, France), *Nlrp3*-/- mice (generated and kindly gifted by Pr Tschopp (University of Lausanne), and *Rorc-/-* mice (purchased from Jackson laboratories - Bar Harbor, USA). C57Bl6 Ly5.1a (referred to hereafter as CD45.1 mice) and OT-I mice (both kindly provided by Pr. Apetoh). The FELASA and the Animal Experimental Ethics Committee of the University of Burgundy, France, Guidelines were followed for the breeding and use of the animals. Six- and 10-week-old mice were used in the experiments.

### T-cell purification, in vitro differentiation and treatment

Mouse naïve CD4+ T cells (CD4^+^CD44^−^) obtained from the spleens and lymph nodes of the mice were purified via a MACS cell separation system (naïve CD4+ cell isolation kit, Miltenyi Biotec). The purity of the isolated naïve T-cell population consistently exceeded 95%. Naïve CD4+ T cells were activated with plate-bound antibodies against CD3 (145-2C11, 2 mg/ml) and anti-CD28 (PV-1, 2 mg/ml) in the presence of specific cytokines for different T-cell lineages: 10 ng/ml of IL-12; 10 µg/ml anti-IL-4 antibody (11B11, BioXcell) for Th1; 10 ng/ml of IL-4; 10 µg/ml anti-IFNγ antibody (XMG1.2, BioXcell) for Th2; 4 ng/ml of TGF-β1; 10 µg/ml anti-IL-4 and 10 µg/ml anti-IFNγ antibodies for Treg; 2 ng/ml of TGF-β; 20 ng/ml of IL-6 and IL-1β; and 25 ng/ml of IL-23 for pTh17; and 20 ng/ml of IL-6 and 2 ng/ml of TGF-β1 for Th17r differentiation. TGF-β3 and IL-6 were also used to induce alternative pTh17 cells. Cytokines were obtained from Miltenyi Biotec. The cells were cultured in RPMI-1640 medium supplemented with 10% (vol/vol) fetal bovine serum (FBS) supplemented with nonessential amino acids (MEM-NEAA), sodium pyruvate, penicillin‒streptomycin/amphotericin B (PSA), and 4 mM 4-(2-hydroxyethyl)-1-piperazineethanesulfonic acid (HEPES).

In the cases described in the text, the cells were pretreated for 1 h before stimulation (or as described in the text if different): LPS (1 µg/ml, Sigma‒Aldrich), ATP (5 mM, Fisher Scientific), Z-VAD (50 µM, Selleckchem), Z-IETD-FMK (50 µM, Selleckchem), MCC950 (10 µM, Merck), anakinra (300 nM, MedChemExpress), 4-hydroxy tamoxifen (1 µM, Sigma Aldrich), SIS3 (10 µM, Merck), LY294002 (325 nM, Selleckchem), SB203580 (10 µM, Selleckchem), Y27632 (10 µM, Seleckchem), JNK Inhibitor II (10 µM, Merck), anti-IFNγ antibody (10 µg/ml, XMG1.2, BioXcell) or recombinant mouse IFNγ (research grade, Miltenyi).

### Flow cytometry

To analyze cells from lymphoid organs (spleen, lymph nodes, and thymus), the organs were dissociated through a 70 µm strainer, washed with PBS, and then labeled.

To analyze TILs, the tumors were cut into small pieces (<0.5 mm) and dissociated via a tumor dissociation kit and a gentleMACS Octo dissociator (Miltenyi). The lysates were filtered through 70 µm strainers and washed with PBS. CD45+ cells were then enriched via CD45 TIL microbeads for mice and magnetic columns (Miltenyi Biotec). After centrifugation, the cell suspensions were incubated for 3 h at 37 °C in RPMI supplemented with 0.2% stimulation cocktail (Cell Stimulation Cocktail plus protein transport inhibitors ref. 00-4975-93 eBioscience). For tumor dissociation, lymphoid organ isolation or culture, the cells were labeled with antibodies against membrane proteins in flow cytometry staining buffer (ref. 00-4222-26 eBioscience). The antibodies used were CD4-V500 (clone RM4-5, BD), CD4-BUV395 (GK1.5, BD), CD45-BUV395 (30-F11, BD), CD45-Viogreen (REA737, Miltenyi), CD8a-PercP-Cy5.5 (53-6.7, BD), CD8a-BUV805 (53-6.7, BD), CXCR3-APC (REA724, Miltenyi), CCR4-PE-Cy7 (2G12, Biolegend), CCR6-BV605 (29-2L17, Biolegend), CD25-AF700 (PC61, Biolegend), CD25-BV (3C3, BD), CD127-BV605 (A7R34, BD), CD39-PE-Cy7 (24DMS1, eBioscience), CD73-PE (TY/11.8, Biolegend), CD45.2-FITC (104, BD), CD44-V450 (IM7, BD), CD62L-BV786 (EL14, Biolegend), and CD107a-PercP-Vio700 (eBio) Then, the fixation and permeabilization of the cells were performed via FoxP3 staining buffer from Miltenyi followed by intracellular staining. The antibodies used were IL-17-APC (51-7177-82, ebBioscience), IL-17-BUV395 (TC11-18H10, BD), IFNγ-BV421 (XMG1.2, Biolegend), Foxp3-PercP-Cy55 (FJK-16s, eBioscience), Foxp3-PE (FJK-16S, Invitrogen), RORγt-APC (AFKJS-9, eBioscience), Ki67-BV605 (16A8, Biolegend), Granzyme B-FITC (REA226, Miltenyi), TNFα-BV510 (MP6-XT22, Biolegend), STAT1pY701-PE (REA159, Miltenyi), STAT3pY705-FITC (13A3-1, Biolegend), pSMAD2/3-PE-CF594 (O72-670, BD), Helios-APC (22F6, Biolegend), and NLRP3-APC (768319, Fisher). All staining included viability staining (Fixable Viability stain, BD). The stained samples were acquired on a Fortessa cytometer (BD), and analyses were performed via FlowJo software (Tree Star, Ashland, OR, USA). Cell sorting was performed with an ARIAII device (BD).

### Cytokine, ATP, AMP, and glycolysis measurements

Secreted cytokines, including IL-1β, IL-18, IFNγ, IL-10, and GM-CSF, were measured via ELISA following the manufacturers’ instructions (BD Biosciences).

For ATP/AMP and glycolysis measurements, the cells were placed in RPMI medium without phenol red (Seahorse XF RPMI, Agilent) supplemented with 10 mM glucose (Seahorse XF Glucose, Agilent), 1 mM pyruvate (Seahorse XF Pyruvate, Agilent) and 2 mM L-glutamine (Seahorse XF L Glutamine, Agilent). For ATP/AMP measurement, the Promega CellTiter-Glo Luminescent Cell Viability assay was used. For glycolysis measurement, the cells were seeded in 96-well test cartridges (Seahorse XF sensor cartridge, Agilent) and centrifuged. Rotenone/antimycin and 2-DG were added to the cartridge according to the manufacturer’s recommendations. The cartridge was then analyzed via the Seahorse XFe96 Analyzer through the Seahorse Glycolytic Rate Assay.

### Cell lines, tumors, and treatments

The J774A.1 cell line (used as a macrophage), B16F10 and LLC1 cell lines obtained from ATCC and B16-OVA kindly provided by Pr Apetoh were maintained at 37°C under 5% CO_2_ in DMEM with 10% FBS supplemented with PSA and 4 mM HEPES.

To induce subcutaneous tumors, 3.10^5^ tumor cells were implanted. Tumor size was regularly monitored every 3 days via a caliper.

For the tumor lung experiments, 2.10^5^ B16-OVA melanoma cells were injected intravenously. Lung tumor foci were counted 13 days after injection.

In some experiments, the following blocking antibodies were administered intraperitoneally (ip) 3 times a week at a dose of 200 µg per mouse: anti-IL-4 (clone 11B11), anti-CD8 (clone YTS169.4), anti-NK1.1 (clone PK136), anti-CD25 (clone PC-61.5.3), anti-TNFα (clone XT3.11), anti-IFNγ (clone XMG1.2) or IgG1 (clone MOPC-21).

Clodronate liposomes, a macrophage inhibitor, were used at 1 mg/mouse, and MCC950, an NLRP3 inhibitor (Merck), at 30 µg/mouse was injected ip twice a week. Digoxin, an RORγt inhibitor (Tocris), was used at 20 µg/mouse per day i.p. Tamoxifen (4 mg/mouse, Bertin Bioreagent) was diluted in sunflower oil and administered daily via oral gavage for 5 days, with pure oil used as a control.

For adoptive transfer experiments, CD4+ T lymphocytes were differentiated as previously described. A total of 2.10^6^ cells were injected per tumor-bearing mouse.

### RNA sequencing

Total RNA from T cells was extracted via TRIzol (Invitrogen, Carlsbad, CA, USA). rRNA was removed via the NEBNext rRNA depletion Kit (NEB, Ipswich, MA, USA). One hundred nanograms of rRNA-depleted RNA was used for library preparation via the NEBNext Ultra II Directional RNA library prep kit for Illumina (NEB) following the manufacturer’s instructions. RNA sequencing was performed on a NextSeq500 device (Illumina, San Diego, CA, USA). The RNA-seq libraries were sequenced with 76 bp paired-end reads. Kallisto software was used to quantify transcript abundance from RNA-seq data against the mm10 version of the *Mus musculus* reference genome. Only protein-coding transcripts and genes were included in the downstream analysis. Differential expression analysis was performed via the DESeq2 R package. The RNA-seq data generated in this study have been deposited in the Gene Expression Omnibus (GEO) database. All other data needed to evaluate the conclusions in the paper are presented in the paper.

### Quantitative PCR analysis

For unfixed cells, total RNA was extracted via TRIzol (Invitrogen, Carlsbad, CA, USA). For fixed cells, RNA was extracted via the Maxwell RSC RNA FFPE Kit (Promega). Quality control was performed via the Agilent RNA ScreenTape Assay on a Tapestation 4200 device (Agilent Technologies). Only RNA with a RIN score of 8 or higher was considered suitable for further analysis. For all the experiments, 300 ng of RNA was reverse-transcribed into cDNA by M-MLV reverse transcriptase, random primers, and RNaseOUT inhibitor (Invitrogen). cDNAs were quantified via real-time PCR with a SYBR Green real-time PCR kit (Applied Biosystems) on a Fast7500 detection system (Applied Biosystems, France). Relative mRNA levels were determined via the 2^-^^ΔΔCt^ method relative to those of β-actin.

### Killing assay and immunosuppressive assay

To assess Th17 cytotoxicity, killing assays were conducted using Th17 cells differentiated for 3 days from either OTII or OTII^*Nlrp3*-/-^ naïve CD4 T cells in the presence of TGF-β and IL-6. These cells were plated in 24-well plates with B16-OVA cells at decreasing ratios of B16-OVA/Th17 for 24 h. Cell viability was determined by flow cytometry after Viab and CD4 staining.

To evaluate CD8+ cytotoxicity, killing assays were performed with CD8+ T cells isolated from B16F10 tumors from CD4^*Nlrp3-/-*^ mice and their littermate controls. The cells were seeded in 24-well plates with B16F10 cells at a 1/1 ratio. Cell death was assessed by flow cytometry through Annexin V/DAPI staining.

Th17 activity was assessed through immunosuppressive assays. Total CD8+ cells were obtained from the spleens and lymph nodes of OTI mice via CD8a (Ly-2) microbeads (Miltenyi). CD8+ cells were cocultured with increasing quantities of Th17 cells differentiated in vitro from OTII or OTII^*Nlrp3-/-*^ naïve CD4+ T cells as previously described in complete RPMI supplemented with the SIINFEKL peptide (2 µg/ml—eBioscience). After 24 h of culture, the cells were harvested, and cytokine production was analyzed via flow cytometry.

### Immunofluorescence (IF) and in situ proximity ligation assay (PLA)

A total of 1 × 10^6^ cells were washed, fixed for 10 min at RT with 4% PFA, and permeabilized for 10 min on ice with 100% glacial methanol. Nonspecific binding was blocked at RT for 1 h with a solution containing 5% FBS and 0.3% Triton X-100 in PBS (for IF). The samples were incubated overnight at 4 °C with primary antibodies (1/100 anti-NLRP3/NALP3, mAb, Cryo-2, Adipogen; 1/50 Smad3 Antibody, Cell Signaling Technology; 1/100 phospho-SMAD3 (Ser423/425), 1/50 anti-TGFβ Receptor Antibody type I, Sigma Aldrich; mouse IgG Isotype Control, Thermo Fisher Scientific; Rabbit IgG Isotype Control, Thermo Fisher Scientific) diluted in a solution of 1% BSA and 0.3% Triton X-100 in PBS. The cells were then washed twice with 0.05% Tween 20 in PBS (PBS-Tween).

For IF, the cells were incubated for 1 h at RT with the secondary antibody diluted in the same buffer. The cells were washed twice with PBS-Tween solution and twice with ultrapure water.

For PLA, the cells were incubated for 30 min at 37 °C with the appropriate probes (Duolink^®^ In Situ PLA^®^ Probe Anti-Rabbit PLUS and Anti-Mouse MINUS; Sigma Aldrich) and for 1 h40 with the polymerase solution following the manufacturer’s recommendations.

For both experiments, stained cells were placed onto microscopy slides (Superfrost Ultra Plus®, Thermo Fisher Scientific) and incubated at RT until the water evaporated. The slides were mounted with a drop of mounting medium containing DAPI (Molecular Probes). Slides were imaged via an Axio Imager—M2 microscope (Zeiss). Images were analyzed by Zen software.

### Western blot and immunoprecipitation assays

Protein extracts were prepared by lysing cells in boiling buffer (1% SDS, 1 mM sodium vanadate, 10 mM Tris [pH 7.4]) in the presence of complete protease inhibitors (Roche diagnostics) for 10 min at 4 °C. The viscosity of the samples was reduced by sonication. The protein concentration was measured via a Bio-Rad DC protein assay kit. Protein lysates were incubated in loading buffer (125 mM Tris-HCl [pH 6.8], 10% β-mercaptoethanol, 4.6% SDS, 20% glycerol, and 0.003% bromophenol blue), heated at 95°C for 5 min, separated by sodium dodecyl sulfate gel electrophoresis (SDS‒PAGE) and electroblotted to nitrocellulose membranes (Schleicher and Schuell). After incubation for 1 h at RT with 5% nonfat milk or BSA in Tris-buffered saline (TBS)-0.1% Tween 20, the membranes were incubated overnight with primary antibody diluted in 5% nonfat milk or BSA in TBS-Tween, washed, incubated with secondary antibody for 45 min at RT, and washed again before analysis with luminol reagent (Santa Cruz Biotechnologies, Heidelberg, Germany).

The mouse monoclonal anti-NLRP3 (Cryo2) antibody was purchased from Adipogen (Liestal, Switzerland); the Smad3 antibody was from Cell Signaling Technology; the phospho-SMAD3 (Ser423/425) and anti-TGFβ receptor type I antibodies were from Sigma Aldrich; the TGF beta receptor 2 (E-6) antibody was from Santa Cruz; and the SARA polyclonal antibody was from Clinisciences. Monoclonal mouse anti-β-actin (A1978) was purchased from Sigma‒Aldrich.

Immunoprecipitation assays were performed with at least 1.10^7^ cells, as previously described [78].

### Chromatin immunoprecipitation assay

Chromatin shearing was performed via a truChIP^TM^ Chromatin Shearing Kit (Covaris) with a focused ultrasonicator M220 device (Covaris). The chromatin immunoprecipitation (ChIP) assay was carried out with a ChIP-IT kit (Active Motif Europe, Rixensart, Belgium) following the manufacturer’s instructions; 1 µg of pSMAD3 antibody (Ser423/425, C25A9, Cell Signaling Technology) or 1 µg of rabbit negative control immunoglobulin G (IgG) was used. DNA precipitation was evaluated via qPCR via the following primers: *Ifng* 5′ccttgggtgtgttgagtgaa3′ and 5′aaaaagccaatgtggtgagg3′, *csf2* 5′ggctactcccatttgactgc3′ and 5′cagcctcagagacccaggta3′, *Tnfa* 5′agggtctgggccatagaact3′ and 5′ccaccacgcttctgtctac3′.

### Statistical analysis

The results are shown as the means ± SDs or SEMs, and the data were compared via the Mann‒Whitney test (test group versus control group) or two-way ANOVA when appropriate. Differences in the number of tumor foci were assessed via one-way ANOVA according to group number. Statistical calculations were performed via GraphPad Prism. All other *p* values were two-tailed. *p*  <  0.05 was considered statistically significant for all experiments.

## Supplementary information

Supplementary figures
