# Supplementary material for: The intrinsic expression of NLRP3 in Th17 cells promotes their protumor activity and conversion into Tregs
Source: Cell Mol Immunol. 2025 Apr 7;22(5):541–56. doi: 10.1038/s41423-025-01281-y (PMC12041534; doi:10.1038/s41423-025-01281-y)
Supplement: Supplementary file 1 — Supplementary figures [file 41423_2025_1281_MOESM1_ESM.pdf]

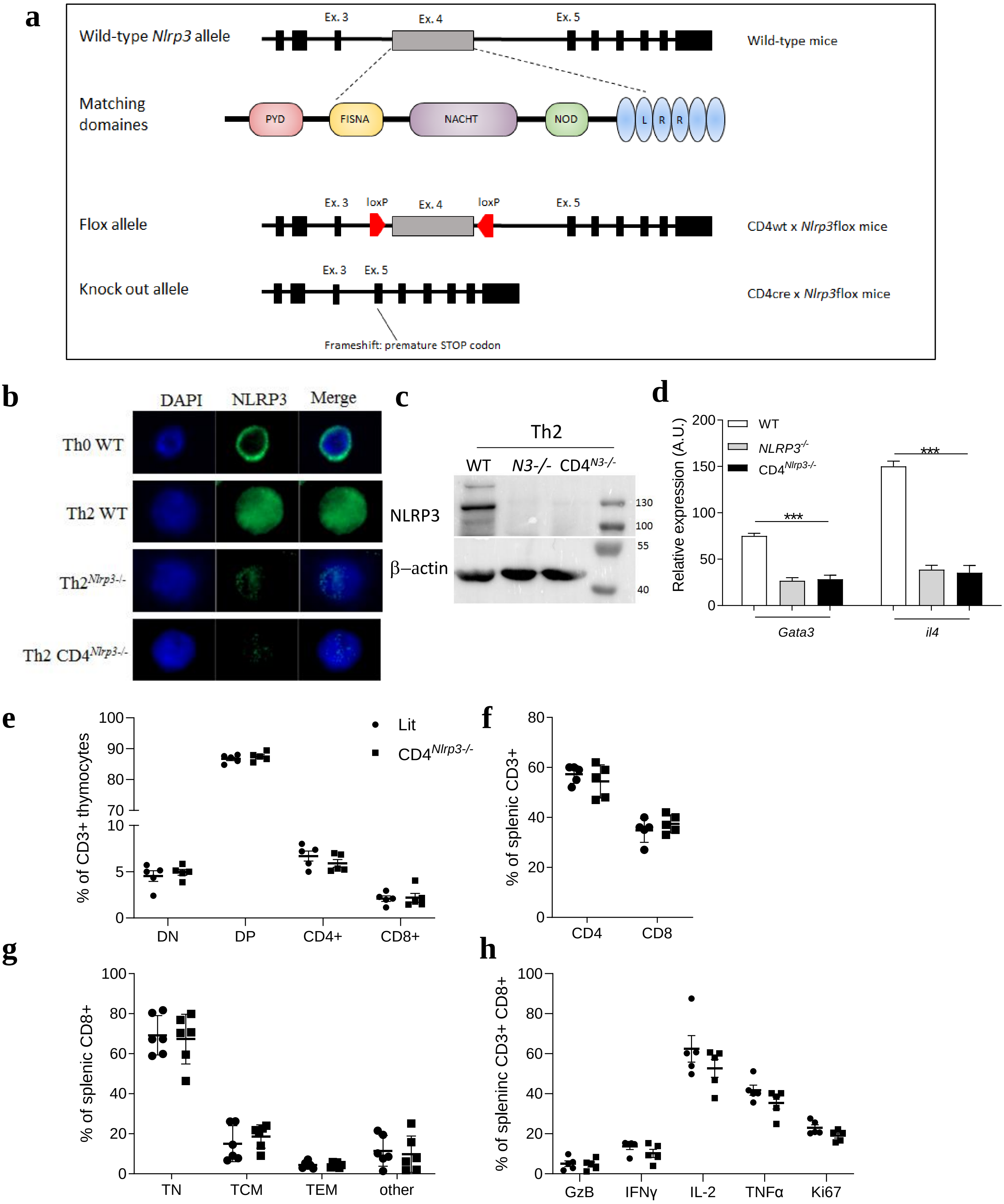
a
Wild-type Nlrp3 allele
Ex. 3
Ex. 4
Ex. 5
Wild-type mice
Matching domaines
PYD
FISNA
NACHT
NOD
L R R
Flox allele
Ex. 3
loxP
Ex. 4
loxP
Ex. 5
CD4wt x Nlrp3flox mice
Knock out allele
Ex. 3
Ex. 5
CD4cre x Nlrp3flox mice
Frameshift: premature STOP codon
b
DAPI
NLRP3
Merge
Th0 WT
Th2 WT
Th2 Nlrp3-/-
Th2 CD4 Nlrp3-/-
c
Th2
WT
N3-/-
CD4 N3-/-
NLRP3
β–actin
130
100
55
40
d
Relative expression (A.U.)
WT
NLRP3-/-
CD4 Nlrp3-/-
***
***
Gata3
il4
e
% of CD3+ thymocytes
Lit
CD4 Nlrp3-/-
DN
DP
CD4+
CD8+
f
% of splenic CD3+
CD4
CD8
g
% of splenic CD8+
TN
TCM
TEM
other
h
% of splenic CD3+ CD8+
GzB
IFNγ
IL-2
TNFα
Ki67

Supplementary Figure 1: Generation and Validation of CD4*^Nlrp3-/-^* Mice. a. Schematic representation of the wild-type, floxed, and knockout *Nlrp3* alleles. Lines = introns; closed boxes = exons; red arrows = loxP sites. **b**. Immunofluorescence microscopy images of Th2 cells polarized from naive CD4+ cells isolated from WT, *Nlrp3-/-*, or $CD4^{Nlrp3-/-}$ mice, after 24 hours of *in vitro* differentiation. Nucleus stained with DAPI (blue) and NLRP3 (green). Representative of 3 independent experiments. **c**. Immunoblot analysis of NLRP3 expression and β-actin as a loading control in Th2 cells polarized as in **b**. **d**. Transcriptional expression of *Gata3* and *Il4* in WT, *Nlrp3-/-*, or $CD4^{Nlrp3-/-}$ Th2 cells polarized as in **b** (n=5). **e**. Frequency of thymocytes expressing CD4, CD8, or double positive (DP) in the thymus of $CD4^{Nlrp3-/-}$ mice and their littermate controls (Lit) (n=5). **f**. Proportions of CD4+ and CD8+ cells among CD3+ splenocytes in the same mice as in **e**. **g**. Frequencies of naive CD8+ (TN), central memory (TCM), and effector memory (TEM) within the CD8+ splenocyte population from the same mice as in **e**. **h**. Frequencies of Granzyme B (GzB), IFNγ, TNFα, and Ki67 expression among CD8+ splenocytes from the same mice as in **e**.
Statistical significance was determined using one-way ANOVA (**d-h**) ***<0.005.

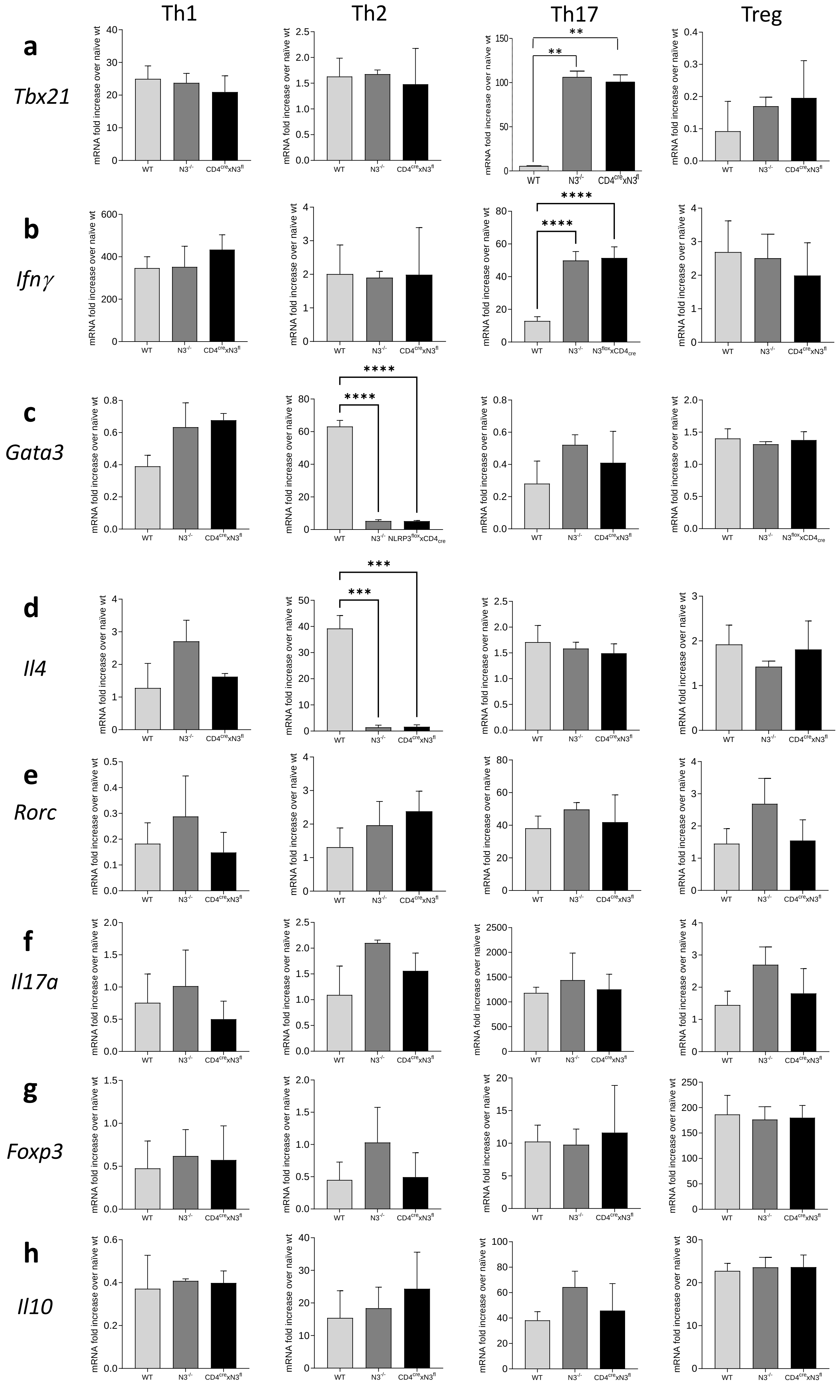

Th1
Th2
Th17
Treg
a
Tbx21
b
Ifnγ
c
Gata3
d
Il4
e
Rorc
f
Il17a
g
Foxp3
h
Il10
mRNA fold increase over naïve wt
WT
N3-/-
CD4crexN3fl
N3floxxCD4cre
NLRP3floxxCD4cre
**
****
***

Supplementary Figure 2: Impact of *Nlrp3* deficiency on the expression of signature genes in T helper subpopulations. mRNA expression of **a**. *Tbx21* **b**. *Ifnγ* **c**. *Gata3* **d**. *Il4* **e**. *Rorc* **f.** *Il17a* **g**. *Foxp3* **h**. *Il10* was measured in Th1, Th2, Th17, or Treg cells after 24 hours of *in vitro* differentiation from naïve CD4+ T cells isolated from WT mice (light gray), fully *Nlrp3*-deficient mice (N3-/-, dark gray), and CD4$^{Nlrp3-/-}$ mice (Nlrp3flox x CD4cre, black). n=5.
Statistical significance was determined using one-way ANOVA ***<0.005, ****<0.001.

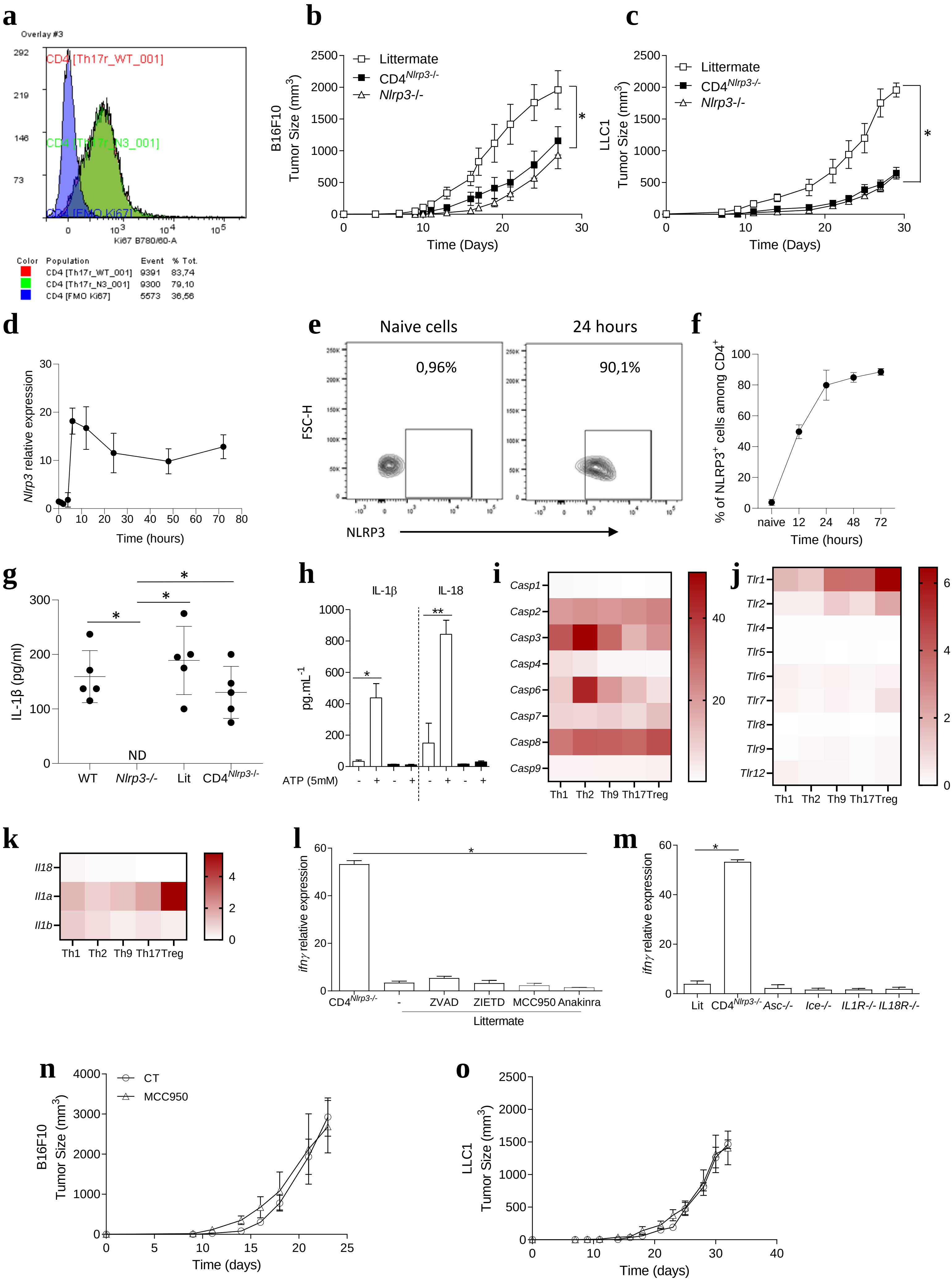

a
Overlay #3
CD4 [Th17r_WT_001]
CD4 [Th17r_N3_001]
CD4 [FMO Ki67]
Ki67 B780/60-A
Color Population Event % Tot.
CD4 [Th17r_WT_001] 9391 83,74
CD4 [Th17r_N3_001] 9300 79,10
CD4 [FMO Ki67] 5573 36,56
b
Littermate
CD4Nlrp3-/-
Nlrp3-/-
B16F10 Tumor Size (mm3)
Time (Days)
*
c
Littermate
CD4Nlrp3-/-
Nlrp3-/-
LLC1 Tumor Size (mm3)
Time (Days)
*
d
Nlrp3 relative expression
Time (hours)
e
Naive cells
24 hours
0,96%
90,1%
FSC-H
NLRP3
f
% of NLRP3+ cells among CD4+
naive 12 24 48 72
Time (hours)
g
IL-1β (pg/ml)
ND
WT Nlrp3-/- Lit CD4Nlrp3-/-
*
h
IL-1β
IL-18
pg.mL-1
ATP (5mM)
*
**
i
Casp1
Casp2
Casp3
Casp4
Casp6
Casp7
Casp8
Casp9
Th1 Th2 Th9 Th17Treg
j
Tlr1
Tlr2
Tlr4
Tlr5
Tlr6
Tlr7
Tlr8
Tlr9
Tlr12
Th1 Th2 Th9 Th17Treg
k
Il18
Il1a
Il1b
Th1 Th2 Th9 Th17Treg
l
ifnγ relative expression
CD4Nlrp3-/-
-
ZVAD
ZIETD
MCC950
Anakinra
Littermate
*
m
ifnγ relative expression
Lit CD4Nlrp3-/- Asc-/- Ice-/- IL1R-/- IL18R-/-
*
n
CT
MCC950
B16F10 Tumor Size (mm3)
Time (days)
o
LLC1 Tumor Size (mm3)
Time (days)

Supplementary Figure 3: The functions of NLRP3 in the inflammasome are not involved in the induction of IFNg in Th17 cells. a. Analysis of cell proliferation by flow cytometry after Ki67 staining in WT or *Nlrp3*$^{-/-}$ naïve CD4+ T cells after 72h of *in vitro* Th17 differentiation (representative of 5 independent experiments). Blue: unstained, Red: WT Th17 cells, Green: *Nlrp3*-deficient Th17 cells. **b and c.** Tumor growth (n=5) of B16F10 (**b**) and LLC1 (**c**) tumors in CD4$^{Nlrp3-/-}$ mice and their littermate controls, as well as in fully *Nlrp3*-deficient mice (*Nlrp3*-/-). **d.** *Nlrp3* relative expression during Th17 differentiation (n=3). **e and f**. NLRP3 protein expression detected by flow cytometry in Th17 cells during differentiation (n=3). **g**. IL-1β production in B16F10 tumors harvested from WT mice, *Nlrp3*-deficient mice (*Nlrp3*$^{-/-}$), CD4$^{Nlrp3-/-}$ mice and their littermate controls (Lit) (n=5). B16F10 tumors were implanted subcutaneously. After 10 days, the tumors were harvested, homogenized, and IL-1β levels were quantified by ELISA. **h**. IL-1β and IL-18 production measured by ELISA in supernatants of macrophages (white bars) or Th17 cells (black bars) treated with 5 mM ATP (+LPS for macrophages) (n=3). **i-k.** Heatmaps of gene expression (reads per kilobase million, rpkm) for genes encoding *Caspases* (**i**), *TLR* receptors (**j**) and select cytokines (**k**) in Th1, Th2, Th9, Th17 and Tregs differentiated *in vitro* for 24h**. l and m**. *Ifnγ* relative expression (n=3) in Th17 cells differentiated from CD4$^{Nlrp3-/-}$ mice and their littermate (Lit) controls treated with CASPASE-1 inhibitor (ZYVAD), CASPASE-8 inhibitor (ZIETD), inflammasome inhibitor (MCC950), or Anakinra (Anak) (**l**) or in Th17 cells derived from mice deficient in *Asc*, *Ice*, *il1ra*, and *il18r* (**m**), **n and o.** Tumor growth of B16F10 (**n**) and LLC1 (**o**) in WT C57Bl6 mice treated or not with the inflammasome inhibitor MCC950 (n=5) .
Statistical significance was determined by 2-way ANOVA (**g**) and Tukey's multiple comparison test (**b, c, n and o**) and by 1-way ANOVA *<0.05, **<0.01 (**h, l m**).

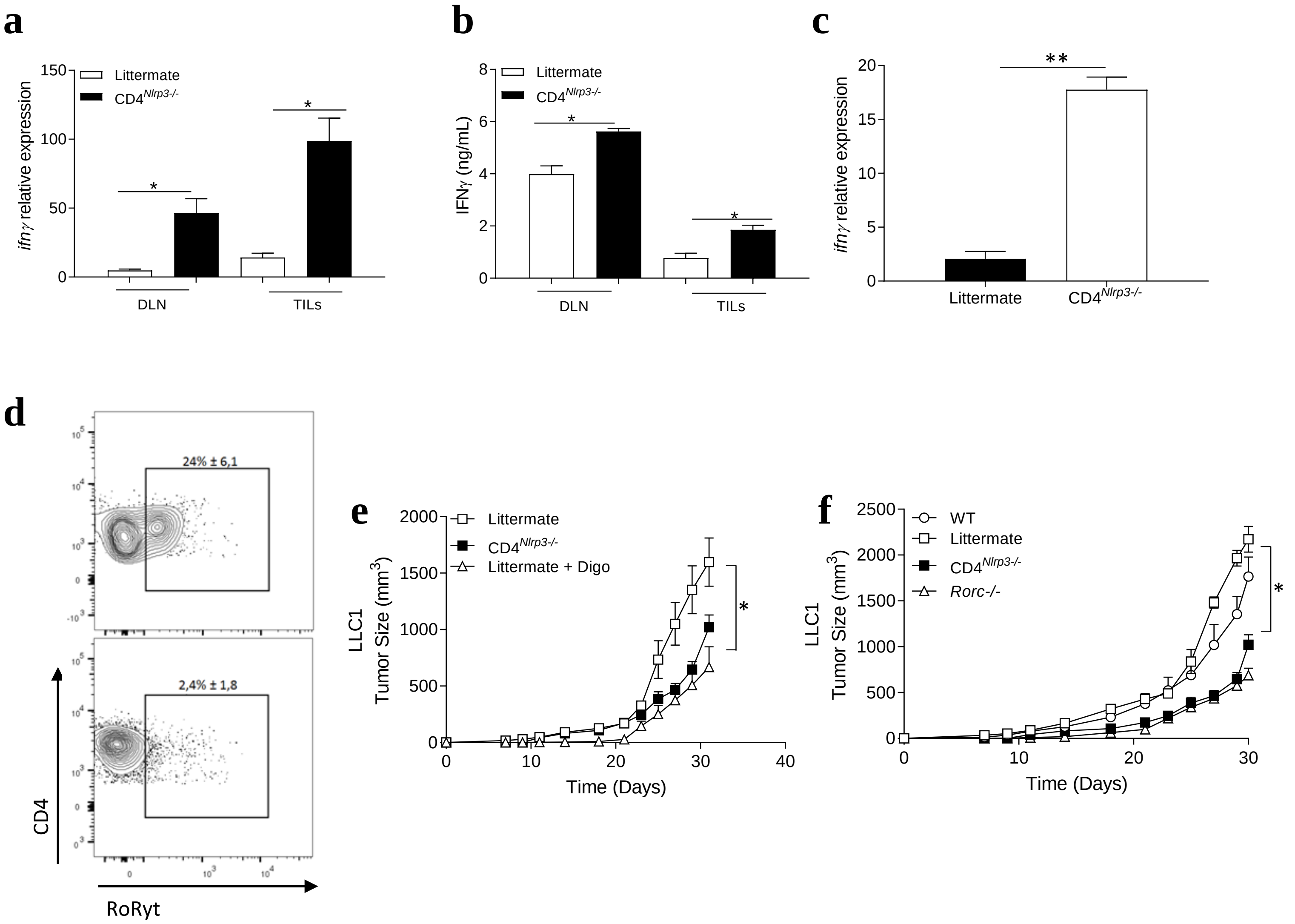

Supplementary Figure 4: Partial switch of regulatory Th17 cells. a. *Ifnγ* relative expression in CD4 T cells isolated from tumor-draining lymph nodes (DLN) and from tumors of $CD4^{Nlrp3-/-}$ mice and their littermate controls (n=5). **b**. IFNγ production evaluated by ELISA in the supernatant of the cells isolated in (**a**) and restimulated over night with anti-CD3 and anti-CD28. **c.** *Ifnγ* relative expression in Th17 cells sorted from tumors of $CD4^{Nlrp3-/-}$ mice and their littermate controls by cytometry as CD4+CCR6+RORγt+Foxp3- (n=5). **d.** Frequency of Th17 cells (identified as RORγt+CD4+ cells) in mice treated daily for 2 weeks with 20 µg/mouse of digoxin, or left untreated. Spleens, lymph nodes, and tumors were harvested, dissociated, stained, and analyzed by flow cytometry. **e and f** LLC1 tumor growth (n=5) in (**e**) $CD4^{Nlrp3-/-}$ and their littermate controls treated or not with Digoxin (Digo) or (**f**) in wild type (WT), *Ror*c-deficient (*Rorc-/-),* $CD4^{Nlrp3-/-}$ and their littermate controls.

Statistical significance was determined by 1-way ANOVA *<0.05, **<0.01 (**a-c**) and by 2-way ANOVA and Tukey's multiple comparison test (**e and f**).

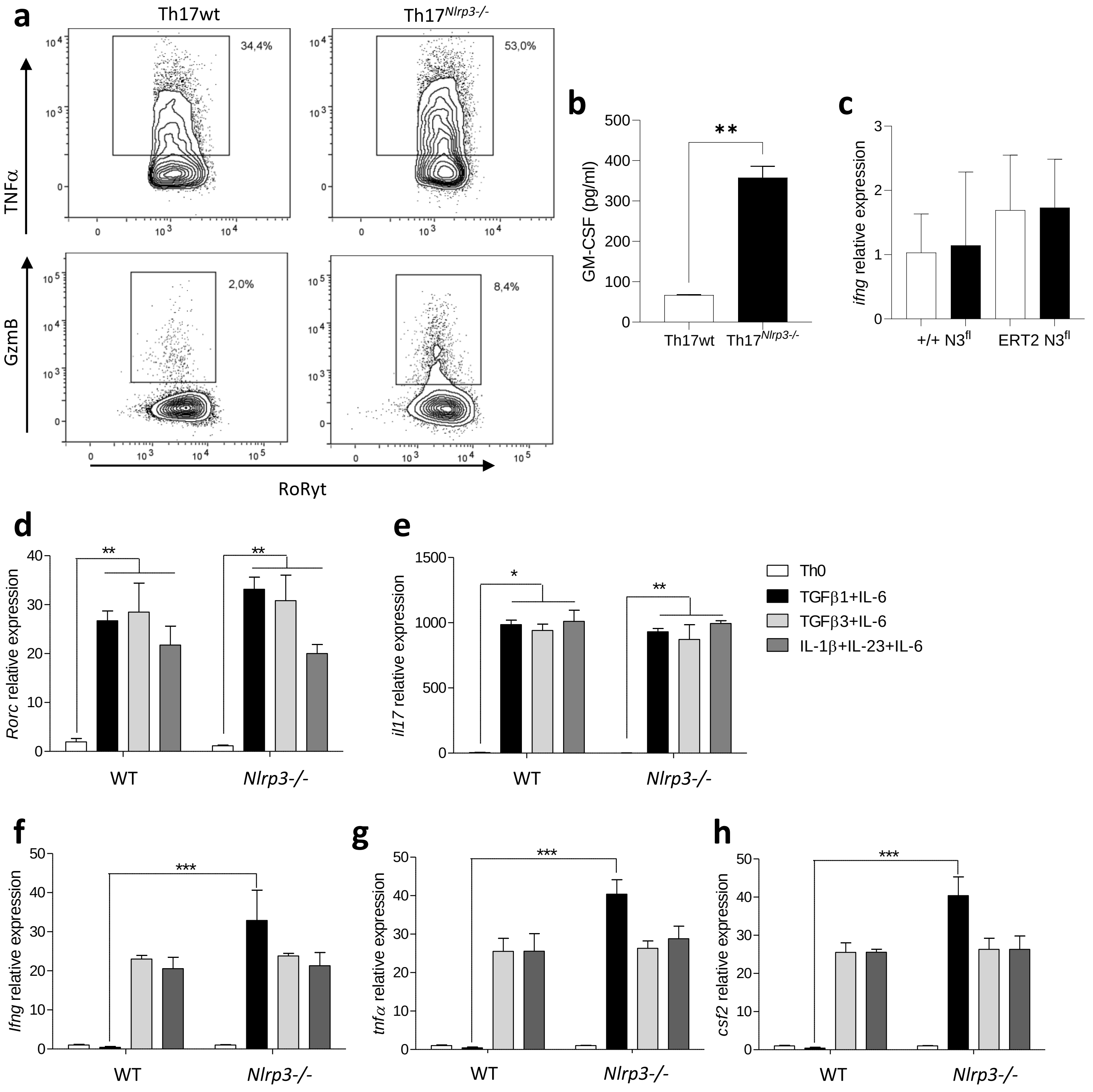

Supplementary Figure 5. Partial switch of regulatory Th17 cells. a-b. Th17 cells (representative of 3 independent experiments) were differentiated for 3 days from naïve CD4+ T cells isolated from CD4$^{Nlrp3-/-}$ mice (Th17$^{Nlrp3-/-}$) and their littermate controls (Th17wt). **a (upper)**. TNFα detection by flow cytometry. **(lower)**. Granzyme B detection by flow cytometry. **b**. GM-CSF production quantified by ELISA. **c**, Relative expression of Ifnγ. Naive CD4 cells were isolated from the spleens of *Nlrp3*$^{flox/flox}$ x CD4^{wt} (+/+ *Nlrp3*-/-) mice and *Nlrp3*$^{flox/flox}$ x CD4^{CreERT2} (ERT2 *Nlrp3*-/-) mice. These cells were differentiated *in vitro* for 72h, followed by 72h treatment with 4-hydroxytamoxifen (black) or vehicle (white). **d-h**. Relative expression of *Rorc* (**d**), *Il17* (**e**), *Ifng* (**f**), *Tnfa* (**g**) and *Csf2* (**h**) in Th0 and Th17 cells differentiated in the presence of TGF-β1 and IL-6, TGF-β3 and IL-6, or IL-1β, IL-23 and IL-6 from naïve CD4+ T cells isolated from WT or *Nlrp3*-deficient mice (n=3).

Statistical significance was determined by a 1-way ANOVA *<0,5, **<0.01, ***<0,001

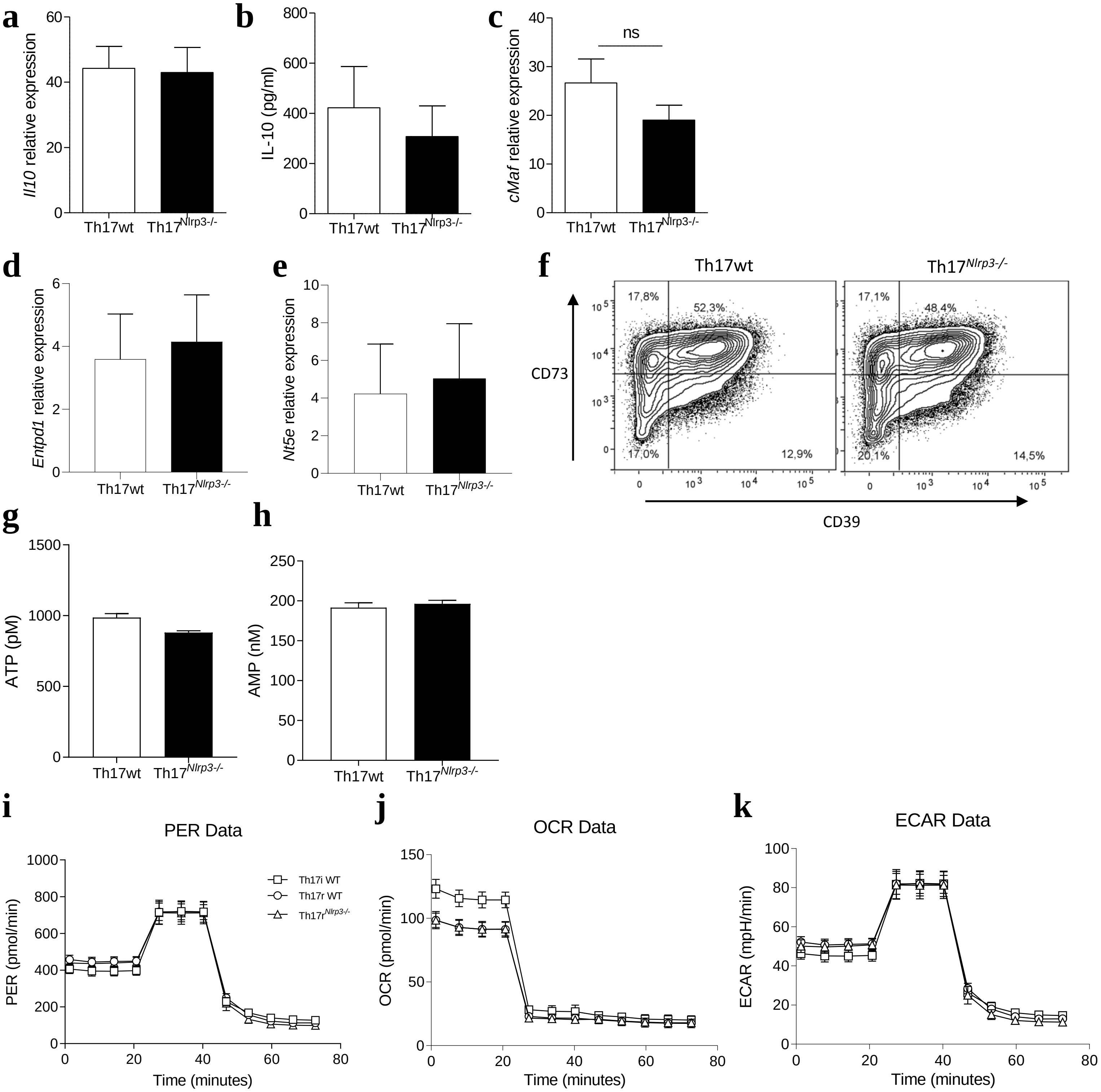

Supplementary Figure 6: Partial switch of regulatory Th17 cells. a-h. Th17 cells were differentiated for 3 days from naïve CD4+ T cells isolated from CD4$^{Nlrp3-/-}$ (Th17$^{Nlrp3-/-}$) mice and their littermate controls (Th17wt) (n=3) **a**. *Il10* relative expression, **b**. IL-10 production quantified by ELISA. **c.** *cMaf* relative expression. **d**. *Entpd1* relative expression. **e**. *Nt5e* relative expression. **f**. Membrane expression of CD39 and CD73 detected by cytometry (representative of 3 independent experiments). **g and h**. Quantity of ATP (**g**) and AMP (**h**) detected in the culture supernatant, **i-k**. Cells were differentiated for 72h *in vitro.* n=3, **i**. Proton efflux rate (PER), **j**. Oxygen consumption rate (OCR) **k**. Extra Cellular Acidification Rate (ECAR) measured over time using an Agilent Seahorse Analyzer, with the addition of Rotenone and Antimycin A (Rot/AA) at 25 min and addition of 2-deoxy-D-glucose (2-DG) at 45 min.
Statistical significance was determined by 2-way ANOVA.

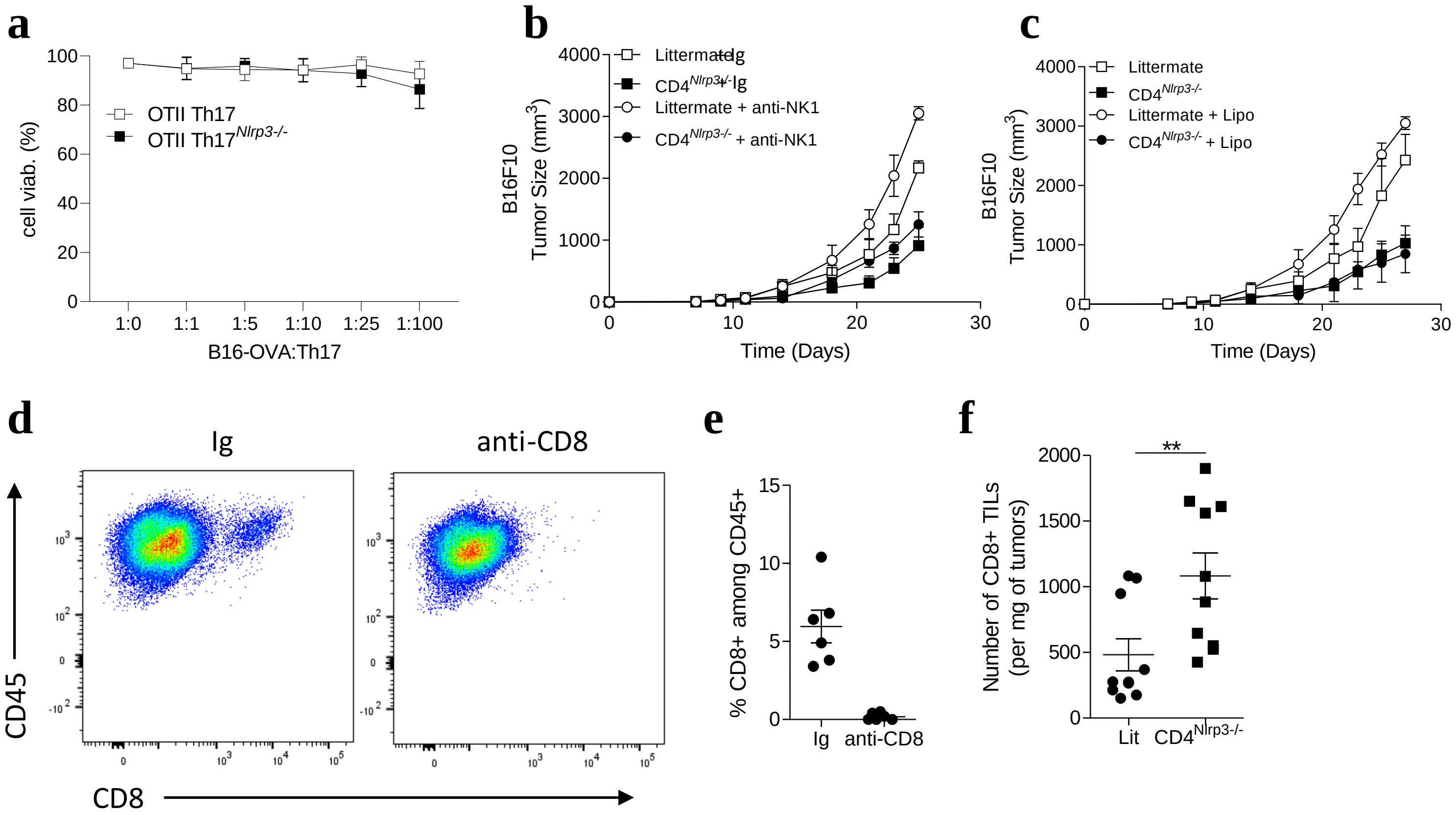

Supplementary Figure 7: Impact of *Nlrp3* deficiency on tumor microenvironment. a. Viability of B16-OVA cells assessed in flow cytometry after 24h of co-culture with Th17 OTII or Th17 OT-II cells deficient in *Nlrp3*$^{-/-}$ cells (n=3), **b, c.** B16F10 tumor growth (n=5) in CD4$^{Nlrp3-/-}$ mice and their littermate controls, treated with either anti-NK1 neutralizing antibody or a control Ig (**b**) and with clodronate liposomes to deplete macrophages (Lipo) or DMSO as vehicle (**c**). **d and e,** Frequency of CD8+ cells (identified as CD45+CD8+ cells) in the tumors of mice treated twice a week with 200 µg/mouse of anti-CD8+ neutralizing antibody or control Ig. **f**, Number of CD8^{+} cells evaluated by cytometry in B16F10 tumors from CD4$^{Nlrp3-/-}$ mice and their littermate controls (Lit). Statistical significance was determined by 2-way ANOVA and Tukey's multiple comparison test (**b and c**) and by 1-way ANOVA *<0.05 (**e and f**).

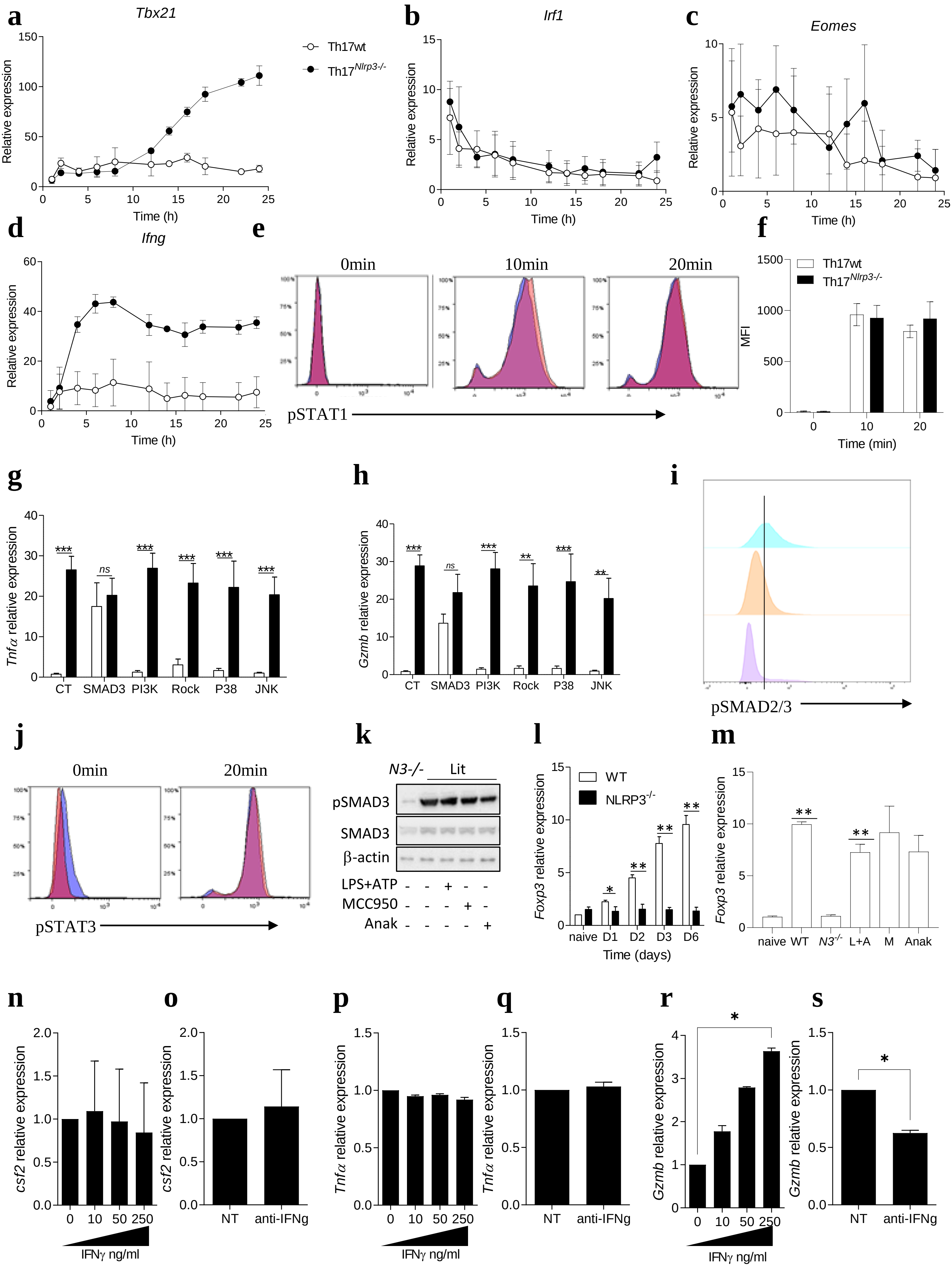

a
Tbx21
Relative expression
Time (h)
Th17wt
Th17Nlrp3-/-
b
Irf1
Relative expression
Time (h)
c
Eomes
Relative expression
Time (h)
d
Ifng
Relative expression
Time (h)
e
0min
10min
20min
pSTAT1
f
Th17wt
Th17Nlrp3-/-
MFI
Time (min)
g
Tnfα relative expression
*** ns *** *** *** ***
CT SMAD3 PI3K Rock P38 JNK
h
Gzmb relative expression
*** ns *** ** *** **
CT SMAD3 PI3K Rock P38 JNK
i
pSMAD2/3
j
0min
20min
pSTAT3
k
N3-/-
Lit
pSMAD3
SMAD3
β-actin
LPS+ATP - - + - -
MCC950 - - - + -
Anak - - - - +
l
Foxp3 relative expression
WT
NLRP3-/-
naive D1 D2 D3 D6
Time (days)
m
Foxp3 relative expression
naive WT N3-/- L+A M Anak
n
csf2 relative expression
0 10 50 250
IFNγ ng/ml
o
csf2 relative expression
NT anti-IFNg
p
Tnfα relative expression
0 10 50 250
IFNγ ng/ml
q
Tnfα relative expression
NT anti-IFNg
r
Gzmb relative expression
0 10 50 250
IFNγ ng/ml
s
Gzmb relative expression
NT anti-IFNg

Supplementary Figure 8: NLRP3 non-canonical functions are involved in Th17 cells. a-d. mRNA expression of *Tbx21* (**a**), *Irf1* (**b**), *Eomes* (**c**) and *Ifng* (**d**), in Th17 WT or *Nlrp3*-deficient Th17 cells during differentiation (n=3). **e and f.** Phosphorylation levels of STAT1 assessed by flow cytometry in naïve CD4+ T cells (0 min) from $CD4^{Nlrp3-/-}$ (orange) and their littermate controls (blue) differentiated into Th17 cells for 10 and 20 minutes (n=3). **g and h**. Relative expression of *Tnf*α (**g**) and *Granzyme B* (**h**) in Th17 cells differentiated from naïve CD4+ T cells from WT (white) or *Nlrp3*-deficient (black) mice and treated with inhibitors of the TGF-β pathway (n=3). **i**. Phosphorylation level of SMAD2/3 assessed by flow cytometry in Th17 cells differentiated from naïve CD4+ T cells from *Nlrp3*-deficient mice (orange) and their littermate controls (blue) for 4 hours (representative of 2 independent experiments, unstained cells in purple). **j**. Phosphorylation level of STAT3 assessed by flow cytometry in naïve CD4+ T cells from $CD4^{Nlrp3-/-}$ (orange) and their littermate controls (blue) differentiated into Th17 (representative of 3 independent experiments). **k**. Naive CD4+ T cells from $CD4^{Nlrp3-/-}$ (*N3*-/-) mice and their littermate controls (Lit) were differentiated *in vitro* into Th17cells. The littermate control cells were simultaneously treated (or not) with LPS+ATP, MCC950, or Anakinra (Anak). After 24 hours, the cells were lysed and analyzed by Western Blot with the indicated antibodies (representative of 3 independent experiments). **l**, Relative expression of *Foxp3* in Th17 cells differentiated from naïve CD4+ T cells from WT or *Nlrp3*-deficient mice over a 6-day period (n=3), **m**. Relative expression of *Foxp3* under the same conditions as **k** (n=3)**. n-s**. n=3, Relative expression of *Csf2* (**n, o**), *Tnfa* (**p, q**) and *Granzyme B* (**r, s**) in Th17 cells treated with increasing doses of IFNγ (**n, p, r**) or with an anti-IFNγ blocking antibody (**o, q, s**).
Statistical significance was determined by 1-way ANOVA *<0.05, **<0.01 (**g-h and l-s**).

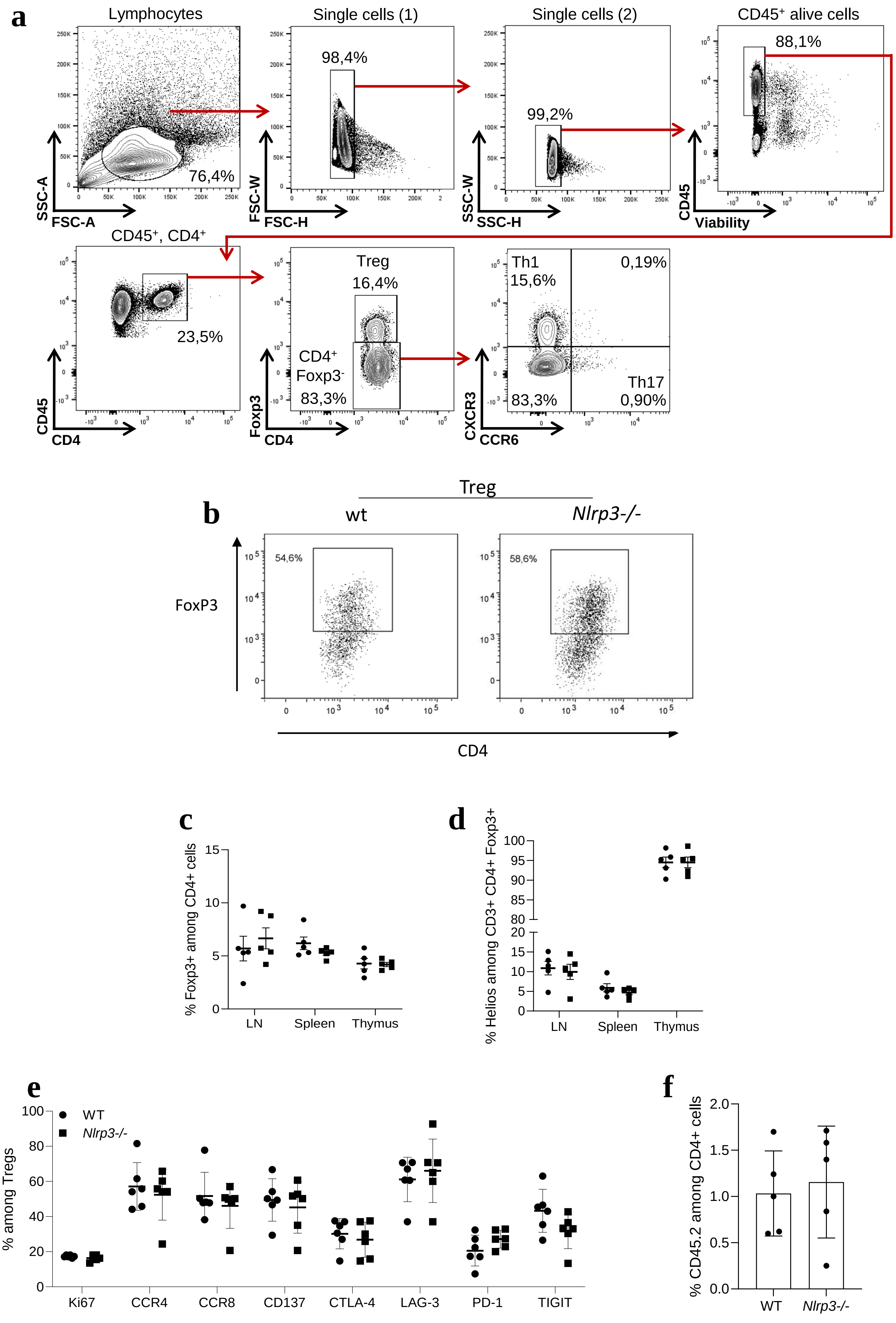

a
Lymphocytes
Single cells (1)
Single cells (2)
CD45⁺ alive cells
76,4%
98,4%
99,2%
88,1%
SSC-A
FSC-A
FSC-W
FSC-H
SSC-W
SSC-H
CD45
Viability
CD45⁺, CD4⁺
23,5%
Treg
16,4%
CD4⁺ Foxp3⁻
83,3%
Th1
15,6%
0,19%
Th17
0,90%
83,3%
CD45
CD4
Foxp3
CD4
CXCR3
CCR6
b
Treg
wt
Nlrp3-/-
FoxP3
54,6%
58,6%
CD4
c
% Foxp3+ among CD4+ cells
LN
Spleen
Thymus
d
% Helios among CD3+ CD4+ Foxp3+
LN
Spleen
Thymus
e
WT
Nlrp3-/-
% among Tregs
Ki67
CCR4
CCR8
CD137
CTLA-4
LAG-3
PD-1
TIGIT
f
% CD45.2 among CD4+ cells
WT
Nlrp3-/-

Supplementary Figure 9: Impact of NLRP3 on Th17 conversion. a. Gating strategy for intra-tumor helper T cell populations. CD4+ T cells ($CD45^+$ $CD4^+$) and subpopulations, Treg ($Foxp3^+$), Th1 ($Foxp3^-$ $CCR6^-$ $CXCR3^+$), Th17 ($Foxp3^-$ $CCR6^+$ $CXCR3^-$). **b.** Proportion of $Foxp3^+$ cells determined by flow cytometry after 72h of *in vitro* differentiation to Tregs differentiated from naïve CD4+ T cells isolated from WT or $CD4^{Nlrp3-/-}$ mice. **c.** Proportion of $Foxp3^+$ cells among CD4+ cells in the Lymph Nodes (LN), Spleen and thymus of $CD4^{Nlrp3-/-}$ (squares) healthy mice and their littermate controls (circles), n=5. **d**. Proportion of $Helios^+$ cells among Foxp3+ cells in the Lymph Nodes (LN), Spleen and thymus of $CD4^{Nlrp3-/-}$ (squares) healthy mice and their littermate controls (circles), n=5. **e**. Proportion of Tregs defined as $CD45+CD4+CD25^{high}Foxp3+$ cells, expressing CTLA-4, CCR4, CCR8, CD137, Lag3, PD1 and TIGIT among B16F10 TILs from $CD4^{Nlrp3-/-}$ (squares) mice and their littermate controls (circles), n=6. **f**. Treg cells were differentiated from naïve CD4+ T cells isolated from CD45.2 OTII WT or *Nlrp3*-deficient mice. These cells were transferred into WT CD45.1 mice bearing B16OVA subcutaneous tumors. Three days later frequency of CD45.2+ cells present in the tumors were analyzed by cytometry among CD4+ T cells (n=5).

Statistical significance was determined by 2-way ANOVA test.

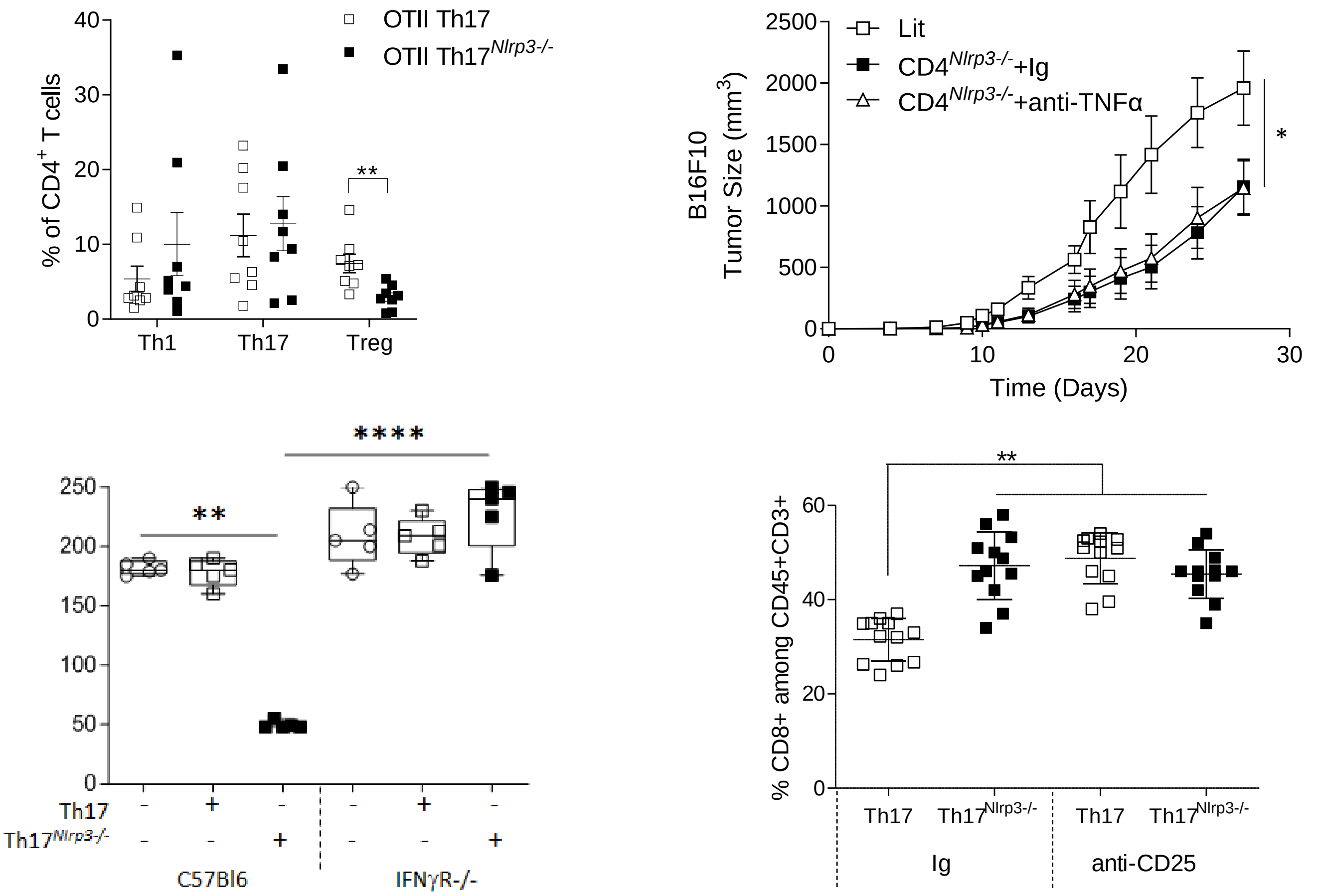

Supplementary Figure 10: Targeting NLRP3 in Th17 cells has therapeutic value, a. Analysis of CD4+ infiltrates in the lungs of mice bearing B16-OVA tumors, 48h after adoptive transfer of CD45.2 OTII Th17 cells or CD45.2 OTII Th17$^{Nlrp3-/-}$ cells. **b**, Tumor growth of subcutaneously injected B16F10 melanoma in CD4$^{Nlrp3-/-}$ mice and their littermate controls. CD4$^{Nlrp3-/-}$ mice were treated twice a week with an anti-TNFα blocking antibody or a control Ig (n=5). **c**, B16-OVA lung tumor foci in C57Bl6 or *IfngReceptor*-deficient mice treated or not with OTII Th17 cells (Th17) or OTII *Nlrp3*-deficient Th17 cells (Th17$^{Nlrp3-/-}$) cells, n=5, **d**. Analysis of CD8+ cells from the lungs of mice bearing B16-OVA lung tumor foci treated or not with OTII Th17 cells (Th17) or OTII Th17$^{Nlrp3-/-}$ cells (Th17$^{Nlrp3-/-}$) in combination or not with an anti-CD25 blocking antibody or control Ig injected twice a week (n=12).
Statistical significance was determined by 1-way ANOVA (**a, c, d**) and Tukey's multiple comparison test (**b**) * <0.05, **<0.01, ***<0.005, ****0.0001.
